# Old school, new rules: floral meristem development revealed by 3D gene expression atlases and high-resolution transcription factor–chromatin dynamics

**DOI:** 10.3389/fpls.2023.1323507

**Published:** 2023-12-13

**Authors:** Margaret Anne Pelayo, Nobutoshi Yamaguchi

**Affiliations:** ^1^ Smurfit Institute of Genetics, Trinity College Dublin, Dublin, Ireland; ^2^ Division of Biological Science, Graduate School of Science and Technology, Nara Institute of Science and Technology, Ikoma, Nara, Japan

**Keywords:** Arabidopsis, chromatin, floral meristem, gene expression, spatial reconstruction, transcription factors

## Abstract

The intricate morphology of the flower is primarily established within floral meristems in which floral organs will be defined and from where the developing flower will emerge. Floral meristem development involves multiscale-level regulation, including lineage and positional mechanisms for establishing cell-type identity, and transcriptional regulation mediated by changes in the chromatin environment. However, many key aspects of floral meristem development remain to be determined, such as: 1) the exact role of cellular location in connecting transcriptional inputs to morphological outcomes, and 2) the precise interactions between transcription factors and chromatin regulators underlying the transcriptional networks that regulate the transition from cell proliferation to differentiation during floral meristem development. Here, we highlight recent studies addressing these points through newly developed spatial reconstruction techniques and high-resolution transcription factor–chromatin environment interactions in the model plant *Arabidopsis thaliana*. Specifically, we feature studies that reconstructed 3D gene expression atlases of the floral meristem. We also discuss how the precise timing of floral meristem specification, floral organ patterning, and floral meristem termination is determined through temporally defined epigenetic dynamics for fine-tuning of gene expression. These studies offer fresh insights into the well-established principles of floral meristem development and outline the potential for further advances in this field in an age of integrated, powerful, multiscale resolution approaches.

## 1 Introduction

Flower architecture is three dimensional (3D), typically being characterized by radial symmetry encompassing a widely conserved basic organ plan, yet is also marked by high phenotypic plasticity across flowering plant species (Endress, 2006; Alvarez-Buylla et al., 2010; Sauquet et al., 2017). Flower morphogenesis underlying floral architecture also occurs in 3D space and over time (adding a dimension, thus 4D) as it progresses across development (Bassel and Smith, 2016). Our understanding of flower formation has been greatly advanced by techniques that have enabled the examination of the floral structure in two dimensions at the cellular level, from inception until the formation of a full flower (Smyth et al., 1990; Reddy et al., 2004; Kwiatkowska, 2006; Bassel and Smith, 2016). The integration of quantitative 2D analyses with the molecular and genetic underpinnings of flower formation can capture the full developmental phenotype in three dimensions (Bassel and Smith, 2016).

In the model plant Arabidopsis (*Arabidopsis thaliana*), early flower development is defined as the period from the floral initiation, or their initial appearance as a floral buttress on the flank of the inflorescence meristem, until buds open as fully formed flowers (Smyth et al., 1990). Detailed examination of this developmental progression has revealed characteristic events that can be sequentially categorized into 12 stages (Smyth et al., 1990; Alvarez-Buylla et al., 2010). Prior to the initiation of the floral meristem, the plant hormone auxin accumulates in the incipient primordium (Heisler et al., 2005; Petrášek and Friml, 2009; Brunoud et al., 2012). This accumulation follows the Fibonacci property of spiral patterns within the shoot apical meristem (SAM), which helps define the positions of consecutive flower primordia (Reinhardt et al., 2003; Heisler et al., 2005; Vanneste and Friml, 2009; Godin et al., 2020). The floral meristem will give rise to all floral organs, namely sepals, petals, stamens, and carpels. Stage 1 is defined by the formation of the floral primordium (floral buttress) on the side of the SAM. By this stage, primordium boundary domains, organ polarity axes, and floral meristem identity are largely specified (Weigel et al., 1992; Talbert et al., 1995; Chen et al., 1999; Heisler et al., 2005; Kwon et al., 2006). Stage 1 can be further classified into substages designated P1 to P6, distinguished by the extent of anisotropic cell growth in the incipient floral primordium (Reddy et al., 2004; Kwiatkowska, 2006; Kwiatkowska, 2008; Alvarez-Buylla et al., 2010). The floral outgrowth will continue to increase in size until stage 2, when a groove forms that separates it from the meristem. The resulting stage-2 flowers contain meristematic regions characterized by an organizing center covered by three distinct cell layers (Mayer et al., 1998; Brand et al., 2000; Daum et al., 2014). Floral organ identity is specified by stage 3 through the classical ABC model by homeotic regulators (Bowman et al., 1991; Parcy et al., 1998; Irish, 2017; Dennis and Peacock, 2019). This model postulates that the floral organs, namely the sepals, petals, stamens, and carpels, arise in their characteristic sequential concentric ring formation through the combinatorial activities of specific genes (Bowman et al., 1989; Scheres, 1998). These genes are classified according to A-, B-, C-type function wherein A genes specify sepals, A and B genes specify petals, B and C genes specify stamens, and C genes specify carpels (Coen and Meyerowitz, 1991; Scheres, 1998). A genes include *APETALA1* (*AP1*) and *APETALA2* (*AP2*) while B genes are comprised of *APETALA3* (*AP3*) and *PISTILLATA* (*PI*), and C gene function is accomplished by *AGAMOUS* (*AG*) (Bowman et al., 1989; Coen and Meyerowitz, 1991; Scheres, 1998; Dennis and Peacock, 2019). Sepal primordia form during stage 3 and continuous sepal outgrowth marks stage 4. The appearance of petal and stamen primordia is next, signifying the start of stage 5. Stage 6 commences when sepals fully overlap the nascent petal and stamen tissues. By this stage, floral meristems have produced sufficient cells to generate all four floral organs, and cell division diminishes (Lenhard et al., 2001; Sun et al., 2009; Liu et al., 2011). In stage 7, stamen primordia form stalks at their base that will give rise to stamen filaments. By stage 8, the formation of anther locules has initiated. Stage 9 is distinguished by an extended period when the petal primordia elongate and become stalked. All floral organs also undergo rapid elongation during this stage. Stage 10 is when the petals are similar in length to that of and reach the top of the lateral stamens. Stigmatic papillae arise during stage 11 and petals reach the height of the medial stamens at stage 12. At the end of stage 12, the sepals open, marking the conclusion of early flower development (Smyth et al., 1990; Alvarez-Buylla et al., 2010). The progression described above has been established by numerous researchers through sectioning or imaging methods using plants grown under various growth conditions, and by investigating the spatiotemporal expression of a handful of key genes in the different floral organs. In order to gain a deeper understanding of flower development and identify additional genes involved in early flower development, including those that are transiently or locally expressed, it is necessary to comprehensively examine spatiotemporal gene expression on a global scale. However, such transcriptome techniques to study flower development in three dimensions remain limited.

The molecular mechanisms regulating floral meristem development is defined by the overarching genetic framework established with the ABC model which has significantly influenced and contributed to the trajectory of flower studies over the years (Bowman et al., 2012; Irish, 2017; Dennis and Peacock, 2019). Through this model, important transcription factors specifying floral cell and tissue identity were identified and characterized, exemplifying essential spatial determinates for gene expression (Praggastis and Thummel, 2017; Thomson et al., 2017; Xu et al., 2021). Proper developmental progression requires coordination between spatial determinants and temporal cues (Praggastis and Thummel, 2017; Xu et al., 2021). Quantifying changes at the cellular, tissue, and organ scales, as well as in gene expression, also requires tracing these events over time (Bassel and Smith, 2016). This can lead to a more accurate understanding of development in four dimensions (which include time). Recent work suggests that the combinatorial binding of key transcription factors to their target genes controls the temporal expression of genes involved in floral transition, floral meristem identity, floral organ identity, and floral organ morphogenesis (Chen et al., 2018). Molecular, genetic, and biochemical analyses have also highlighted the importance of epigenetic mechanisms in finely regulating the timing of gene expression by key transcriptional regulators (Pelayo et al., 2021). In particular, a significant breakthrough in understanding temporal epigenetic mechanisms was achieved with the establishment of a synchronization system for floral development in Arabidopsis (Wellmer et al., 2006). This system uses the *apetala1 cauliflower* (*ap1 cal*) double mutant that produces a characteristic cauliflower phenotype and carrying an AP1 transgene fused to the glucocorticoid receptor (GR) (*ap1 cal proAP1:AP1-GR*). This enables production of AP1-GR protein that localizes in the cytosol. When *ap1 cal proAP1:AP1-GR* plants are treated exogenously with the steroid hormone dexamethasone (DEX), AP1-GR is relocated to the nucleus and can initiate synchronous flower development. This allows researchers to easily prepare and work with floral stage-specific tissues for epigenetic analyses (Smaczniak et al., 2012; Pajoro et al., 2014; Engelhorn et al., 2018). While our knowledge is still limited, the dynamics of epigenetic regulation linked to transcription factors during floral meristem development is starting to emerge.

In this review, we discuss recent advances in how to study floral meristem development in four dimensions, using the fourth dimension (time) as a marker of developmental progression. We provide a broad overview of floral meristem development in the context of newly developed 3D spatial-reconstruction approaches, which address gaps in our understanding between transcriptional inputs and morphological outcomes. Following this overview, we present studies tackling the temporal control of transcription during floral meristem development and propose that the timing of flower formation is epigenetically regulated.

## 2 Floral meristem development in 4D: Spatiotemporally resolved gene expression in floral meristems and epigenetic regulation of timing floral meristem development

### 2.1 Spatial localization of transcripts and reconstruction of their expression domains in Arabidopsis flowers

#### 2.1.1 Spatial detection of RNA and mapping of gene expression domains in Arabidopsis flowers

**Figure 1:**
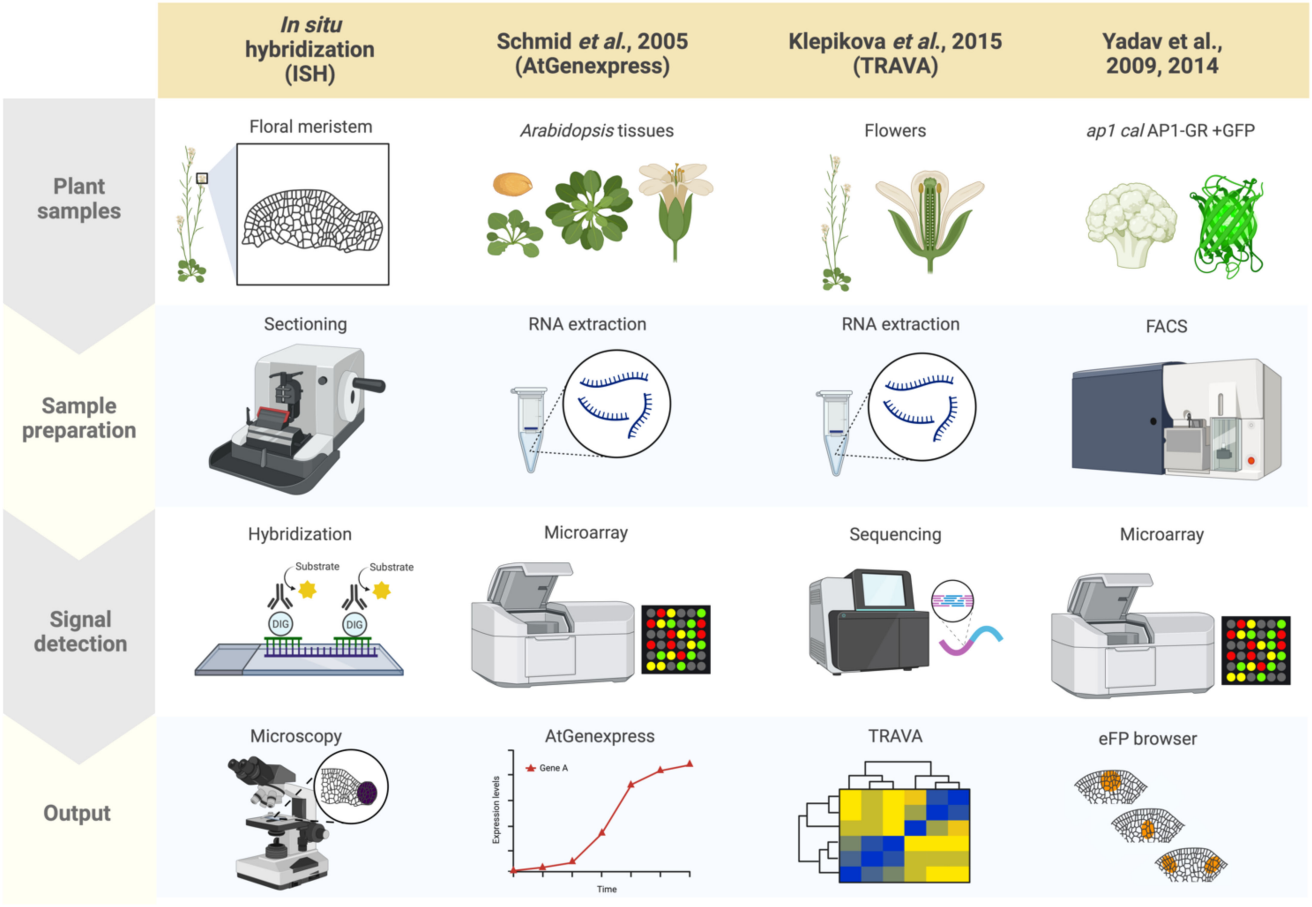
Summary of plant spatial RNA detection approaches. Four plant spatial RNA detection approaches are described, as indicated in the columns: *In situ* hybridization (ISH), AtGenExpress (Schmid et al., 2005), TRAVA (Klepikova et al., 2016), and the FACS-based early flower profiling by Yadav et al. (2009); Yadav et al. (2014) available on the eFP browser. Each row indicates which tissues are used for each approach (‘Plant samples’), the techniques used for preparing the samples (‘Sample preparation’), the method applied for detecting RNA (‘Signal detection’), and how the results from each approach were visualized and summarized (‘Output’). ISH involves preparing tissue sections from plant structures of interest (such as floral meristems for this review) followed by hybridizing probes specific to target mRNA, then visualization through microscopy techniques. AtGenExpress was developed using tissues derived from all Arabidopsis organs at various stages of development subjected to RNA extraction for microarray analysis, generating global gene expression patterns. TRAVA is an RNA-seq-based transcriptome database initially developed for Arabidopsis floral initiation. Lastly, the FACS-based early flower dataset was generated by fluorescently labeling protoplasts of *ap1 cal* plants with an inducible *AP1-GR* transgene, enabling cell-type-specific microarray analysis that was integrated onto the eFP browser.

In plants, as well as in other eukaryotes, established and standard techniques for mapping gene expression to specific cells and tissues typically involves *in situ* hybridization, during which labeled RNA probes complementary to a transcript of interest are hybridized onto local mRNAs in a given sample, revealing the spatial context of target transcript accumulation upon visualizing the labeled samples (**Figure 1**) (Jensen, 2014; Waylen et al., 2020). Samples can be visualized using radioisotope- or non-radioisotope-labeled RNA probes (Houben et al., 2006). In particular, digoxigenin-labeled RNA probes allow for higher resolution than radioisotope-based methods (Wu and Wagner, 2012). Furthermore, *in situ* techniques have been adapted to accommodate a wide range of sample types, from thin sections mounted onto slides to whole organs and tissues. Aspects of flower development that have been characterized using *in situ* hybridization include the expression patterns of the floral organ identity genes and the transcripts encoding their interactors (Yanofsky et al., 1990; Drews et al., 1991; Bowman et al., 1992). Although it is possible that cross-hybridization may occur between ribonucleic acids derived from orthologous or paralogous genes, gene expression patterns based on *in situ* hybridization and reporter assays (i.e. promoter-reporter fusions with GUS or GFP) are largely identical to each other (Peiffer et al., 2008). Because generating transgenic plants and crossing the transgene into different backgrounds (e.g., mutant backgrounds) is not required, *in situ* hybridization remains a powerful method for obtaining spatial information about RNA molecules (Hu et al., 2023). A major limitation of *in situ* hybridization is its low throughput.

The advent of approaches that dissect transcriptome dynamics, particularly microarrays and transcriptome deep sequencing (RNA-seq), have empowered the quantification of gene expression levels in a high-throughput manner. AtGenExpress is an international project that summarized large-scale microarray data in Arabidopsis (**Figure 1**) (Schmid et al., 2005). The database included gene expression data for many developmental stages as well in response to environmental stimuli. Due to the limitation of probes for microarrays, the number of genes that can be detected is limited, which is not the case with RNA-seq, which provides access to virtually all RNA molecules in a sample. One useful database summarizing gene expression profiles based on RNA-seq data, primarily using floral tissues, is TRAnscriptome Variation Analysis (TRAVA) (**Figure 1**) (Klepikova et al., 2016; Klepikova et al., 2019). TRAVA provides genome-wide floral organ- or stage-specific gene expression levels. In both databases, the resolution of gene expression is at the level of entire organs, not cells.

By combining RNA extraction or nuclei isolation from specific cell types with transcriptome methods, many researchers have sought to provide spatial gene expression information at the tissue-specific or cell-type levels. Such specific cell populations are often collected by fluorescence-activated cell sorting (FACS), isolation of nuclei tagged in specific cell types (INTACT), or laser microdissection (LSM) (Casson et al., 2005; Hölscher and Schneider, 2008; Deal and Henikoff, 2011; Galbraith, 2014). For example, FACS has been used to map gene expression in Arabidopsis inflorescence and floral primordia. Although FACS requires costly equipment and trained personnel, cells prepared from Arabidopsis inflorescences are morphologically uniform and easily sorted making FACS a valuable technique. Furthermore, Yadav et al. (2009); Yadav et al. (2014) employed fluorescence markers expressed in stem cells, three distinct cell layers, or early floral primordia in inflorescence meristems, followed by FACS and subsequent microarray analysis of the extracted RNA (**Figure 1**). Based on the obtained cell type-specific gene expression profiling, a high-resolution gene expression map has been established and made available to the public through the Arabidopsis eFP Browser (Yadav et al., 2009; Yadav et al., 2014). One downside is that since FACS requires protoplast isolation, which typically takes several hours, the resulting expression profile may not accurately reflect true spatial RNA levels or distributions.

#### 2.1.2 Spatial transcriptomes and mapping of gene expression domains in Arabidopsis flowers

**Figure 2:**
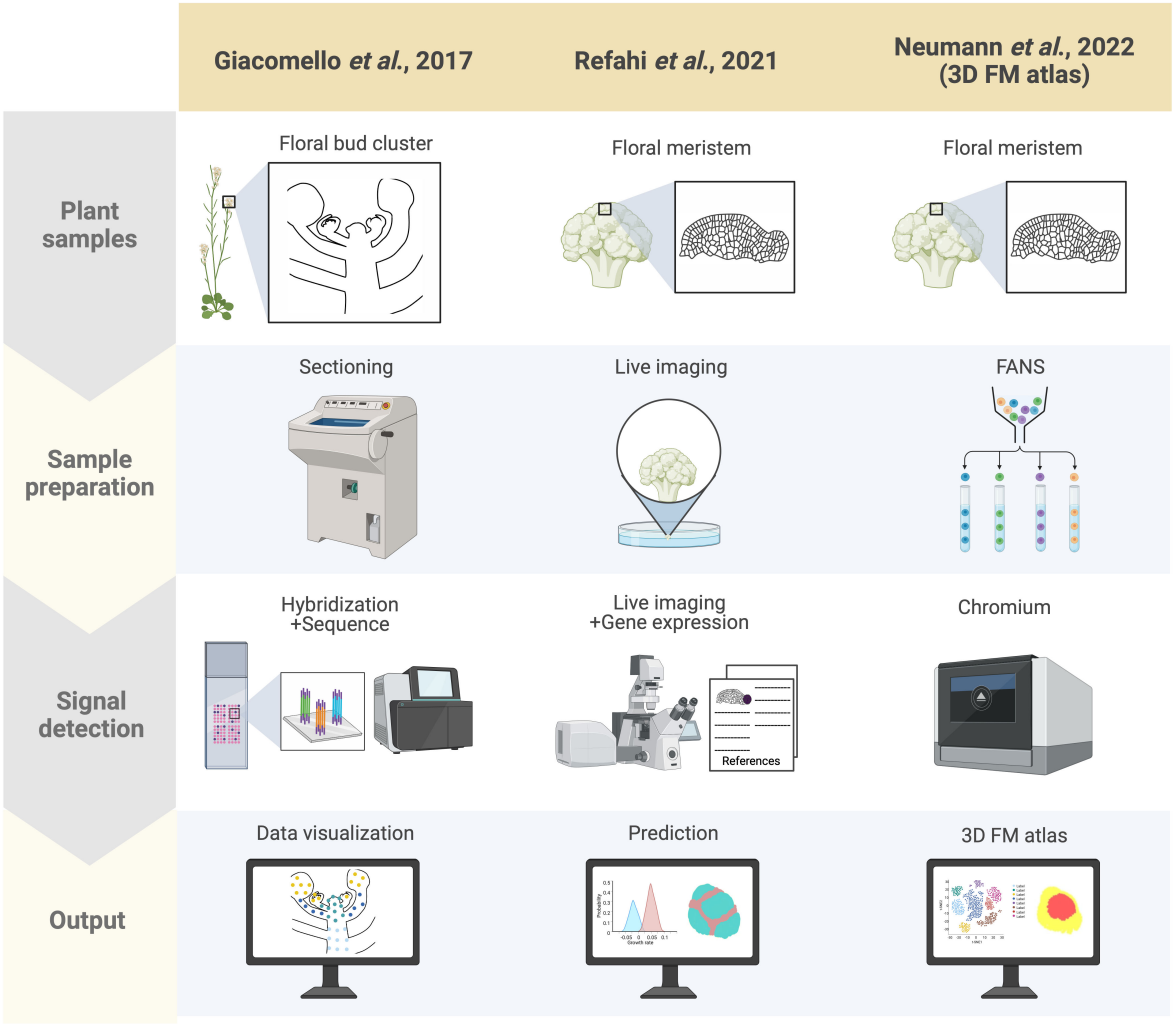
Summary of spatially resolved flower transcriptomes. Three spatially resolved flower transcriptomes are currently available, generated by different groups indicated in the columns: the spatial transcriptomics-based dataset by Giacomello et al. (2017), the live imaging and ISH-based 4D atlas by Refahi et al. (2021), and the scRNA-seq-based 3D atlas by Neumann et al. (2022). Each row indicates which samples were used for each approach (‘Plant samples’), the techniques used for preparing the samples (‘Sample preparation’), the method applied for detecting RNA (‘Signal detection’), and how the results from each approach were rendered (‘Output’). Here signal detection involves both the transcriptional readout and its associated morphological coordinates with the ultimate aim of integrating these two factors in the output. For the adapted spatial transcriptomics by Giacomello et al. (2017), cryo-sections of Arabidopsis inflorescences were prepared, followed by barcoded probe hybridization, wherein the plant sections are laid onto an immobilized spot array containing barcoded oligonucleotide probes, which enables spatial localization of transcripts. Addition of fluorescent labeling during cDNA synthesis also enables morphologically defined tissue-specific transcript visualization. The early flower 4D atlas by Refahi et al. (2021) was established by integrating time-course live imaging of the early flower with curated literature-based gene expression data with their own ISH data projected onto MorphoNet. Finally, the 3D floral meristem (FM) atlas by Neumann et al. (2022) was generated using FANS-based scRNA-seq data mapped onto the 4D atlas of Refahi and colleagues by adapting NovoSpARc, a computational framework for gene expression cartography based on probabilistic optimization matching (Nitzan et al., 2019; Moriel et al., 2021).

**Figure 3:**
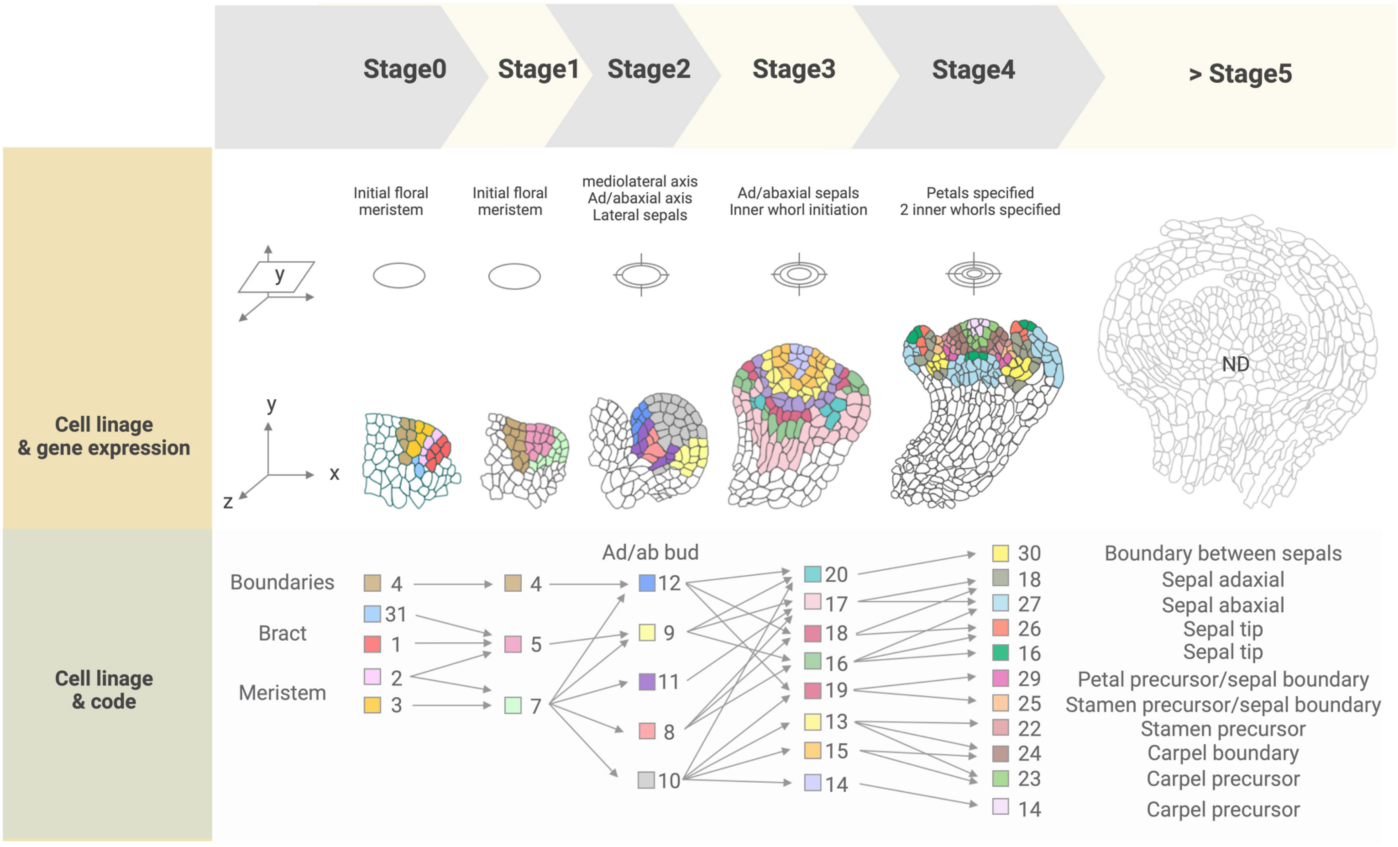
Cell lineage and gene expression tracking of floral stages 0 to 4 from the 4D FM atlas. ‘Cell states’ present in each stage are indicated by a color-coded numbered legend (‘Code’) and can be spatially visualized and tracked over the course of development. Expressed genes included in the atlas (‘reference genes’) are listed in **Supplemental Table 1**, while combinations of these genes expressed in each cell state are listed in **Supplemental Table 2**. Cell states were not described for flower stages after stage 4, indicated as 'ND' (>Stage 5).

Spatial transcriptomics methods detect the positional context of transcriptional activity in intact plants at a higher resolution than previously available approaches, either for regions or single cells. Spatial transcriptomics was initially developed for mammalian systems, using positional barcodes on an oligonucleotide array for RNA-seq on fixed mouse (*Mus musculus*) brain and human (*Homo sapiens*) breast cancer tissue sections (Ståhl et al., 2016; Waylen et al., 2020). In plants, the technique has been applied to the Arabidopsis inflorescence, European aspen (*Populus tremula*) developing and dormant leaf buds, and Norway spruce (*Picea abies*) female cones, representative species of angiosperms and gymnosperms, and to demonstrate the adaptability and reproducibility of this technique across a wide range of plant sample types (**Figure 2**) (Giacomello et al., 2017). The spatial transcriptome data from this study showed the distinct gene expression patterns across different tissue domains within an organ. Furthermore, their data correlated well with the available AtGenExpress-based microarray data. The coming of age of single-cell RNA-seq (scRNA-seq) has also advanced the high-throughput detection of spatially resolved gene expression. Researchers sought to enhance the potential of scRNA-seq by integration with imaging capabilities (Marx, 2021). Few studies have reconstructed spatial gene expression models of early flowers by integrating established techniques such as *in situ* hybridization and confocal imaging with high-throughput transcriptomic approaches to recapitulate early flower development in four dimensions. Nevertheless, Refahi et al. (2021) aimed to establish a direct and quantitative link between organ formation, growth patterns, and their underlying gene expression networks (**Figure 2**). The group initially established a confocal imaging-based time-series cell lineage tracking system for floral meristems by analyzing floral meristem stages based on overall meristem shape. Their focus was primarily on the initiation phase up to stage 4 due to the high consistency and reproducibility observed in their experiments. The resulting 4D template was integrated with expression patterns from 28 well-characterized genes associated with floral meristem development by manually annotating literature-based data and new *in situ*-based data. *In silico* analyses revealed various ‘cell states’ resulting from unique combinations of expressed genes in cell groups and the particular differentiation state of the cell groups (**Figure 3**). The integrated 4D template enabled the researchers to assess correlations between lineage-based data and literature-based data resulting in the enhanced predictive ability of gene expression, assembled into a template. Using this template, the switch to heterogenous growth that takes place at flower stage 2 was identified by quantitative analysis of cellular properties (defined as cell size distribution and number of neighboring cells within the L1 and L2 layers), growth rates, and growth anisotropy (defined by relative growth rates and directionality between successive time points). Moreover, gene expression could be correlated with growth patterns through pairwise comparisons of genes with partially overlapping expression patterns. Using this approach, genes such as *CUP-SHAPED COTYLEDON1–3* (*CUC1–3*), known for their role in proper SAM formation and for proper organ separation between cotyledons and floral organs (Takada et al., 2001), and *ARABIDOPSIS HISTIDINE PHOSPHOTRANSFER PROTEIN6* (*AHP6*), a negative regulator of cytokinin signaling (Besnard et al., 2014), were confirmed for their growth inhibiting and promoting effects, respectively. Finally, the authors established a role for LEAFY (LFY) in regulating growth coordination across cell domains by demonstrating that a strong loss-of-function *lfy* allele showed a slower growth rate in domains in which *LFY* was expressed compared to the wild type. Overall, this work represents an integrated multi-scale analysis of spatiotemporal gene expression patterns during floral meristem development, enabling quantitative correlations between transcriptional inputs and growth outcomes, as well as providing an adaptable and predictive system for analyzing cellular spatial gene expression.

A high-throughput single-cell-based 3D floral meristem atlas was established by Neumann et al. (2022) by adapting NovoSpaRc, a computational framework or algorithm for gene expression mapping based on probabilistic optimization matching, to integrate single nuclei RNA-seq (snRNA-seq) data onto Refahi et al.’s 3D floral meristem atlas (**Figure 2**) (Nitzan et al., 2019; Moriel et al., 2021; Refahi et al., 2021; Neumann et al., 2022). Data integration with NovoSpaRc takes into account transcriptome similarities in two ways, 1) cell-to-cell transcriptome similarity among adjacent cells on the reference map (similarity score between cells within the scRNA-seq/snRNA-seq data as distributed onto the reference map) and 2) similarity score between the scRNA-seq/snRNA-seq dataset and the cells they are assigned to on the reference map (Nitzan et al., 2019; Moriel et al., 2021; Neumann et al., 2022), making it an ideal approach for organisms or any biological system with a limited set of reference genes available. In this study, the authors highlighted the importance of the spatial context of gene expression dynamics in uncovering the molecular networks behind cellular differentiation during development (Neumann et al., 2022). First, they established a snRNA-seq dataset using fluorescence-activated 4’,6-diamidino-2-phenylindole (DAPI)-stained nuclei sorting (FANS) for nuclei collection from floral tissues at stages 4 and 5 derived from the synchronized *ap1 cal proAP1:AP1-GR* inducible system. The snRNA-seq data formed 12 clusters defined by the top 20 marker genes specific to each cluster. By taking these marker genes and plotting their expression against publicly available bulk flower RNA-seq datasets, many of the clusters were assigned to tissue domains (epidermal and vasculature) and cell cycle status. The resulting snRNA-seq dataset was mapped onto the 4D template by Refahi et al. (2021) using a modified NovoSpaRc approach to account for factors such as tissue sample integrity of the snRNA-seq dataset and binary 4D template. They showed that a predicted estimation performance for each gene can be determined prior to establishing the reference spatial map, enabling the assessment of which reference genes can be reliably used for predicting spatial gene expression for targets of interest. Time course analysis with the integrated map indicated that gene expression programs in later flower stages are primed during early flower development, and also demonstrated that this approach can be used to investigate precise spatiotemporal gene expression dynamics. Furthermore, they showed that gene expression levels within specific floral meristem domains (such as the *AP3* and *AG* domains) can be reliably estimated with the reconstructed spatial gene expression map of the floral meristem, as well as for predicting domain-specific differential gene expression, especially relating to floral whorl-specific genes. Notably, they found that gene expression patterns from snRNA-seq data were better represented when mapped onto a spatial reference (as opposed to snRNA-seq data directly compared to bulk RNA-seq data), indicating that integrating snRNA-seq datasets with spatial gene expression maps is a key consideration for conducting multi-scale studies. Lastly, the authors also investigated the establishment of vascular stem cells in the apical meristems to expand on their finding of floral meristem domains associated with vascular-specific marker genes. They used previously characterized transcriptomic data from vascular tissue of inflorescence stems to predict the location of vascular cell initiation in the reconstructed 3D meristem utilizing *SMAX1-LIKE 5* (*SMXL5*) and *PHLOEM INTERCALATED WITH XYLEM* (*PXY*) as markers for the distal cambium and the proximal cambium, respectively (Shi et al., 2021; Neumann et al., 2022). Through this approach, they demonstrated that the spatially reconstructed data could be used to predict the cellular localization of morphologically indistinguishable cells (the distal cambium and the proximal cambium in this case).

Spatial transcriptomics and 3D imaging methods for plants are continually evolving and advancing. For example, an updated approach to investigating spatial gene expression in plants at single-cell resolution was recently described, called PHYTOMap, wherein cell-type markers were spatially detected simultaneously in cleared whole-mount root tissues (Nobori et al., 2023). PHYTOMap overcomes the limitations in sectioning of tissue samples (for organs that are too small to be sectioned, such as root tips), such as is typically used for spatial transcriptomics, making it a viable option to spatially map gene expression in a wide array of tissue types. Studies directly addressing issues in imaging analysis and software development for 3D atlases of ovules have also been recently published (Vijayan et al., 2021; Vijayan et al., 2022). The 2021 study featured a quantitative 3D reference atlas of the Arabidopsis ovule, laying out a framework for morphogenetic characterization of deep tissue structures (Vijayan et al., 2021). The 2022 study is on the use of 3DCoordX, a cell location annotation tool in the open-source visualization and analysis software MorphoGraphX for 4D biological datasets, for precisely and rapidly annotating cells in 3D organ-level representations at single-cell resolution (Vijayan et al., 2022). Both studies will be useful resources for integration with high-throughput gene expression analysis approaches. These approaches demonstrate that spatial gene expression in plants and 3D digital representations of these expression patterns can be scaled globally and can be adapted for different plant tissue types, including floral meristems.

Moreover, spatially resolved transcriptome atlases at single-cell resolution are expected to contribute novel findings not only in studies investigating cell fate reprogramming and lineage mechanisms but also in understanding plant-environment interactions, such as during plant abiotic and biotic stress responses (Chen et al., 2023; Yin et al., 2023). Thus, establishing gene expression atlases is important for all plant scientists engaged in either fundamental or applied research. Ideally, these techniques can be adapted for any area of plant studies and plant system of interest. Additionally, spatially resolved multi-omics approaches beyond the transcriptome level are also currently being developed and adapted to achieve single-cell, or at least, near single-cell, resolution. These include spatially resolved epigenomics, proteomics, and metabolomics (Yu et al., 2022; Yin et al., 2023) techniques. The ultimate aim is to establish and integrate multi-omics atlases and to recapitulate these cellular processes simultaneously in their native spatiotemporal context.

Overall, recent advances in high-throughput single-cell transcriptomic analyses, powerful computing methods, and imaging technologies are contributing to the growth of 3D spatial reconstruction in the plant community. These advances are enabling integrated and quantitative 3D representations of complex structures (such as the flower) at an unprecedented resolution and scale, providing further insight into well-characterized growth and development. These emerging approaches will undoubtedly serve as an important gateway in establishing testable and quantifiable links between the regulation of gene expression and morphogenesis.

### 2.2 Temporal control of transcription in the floral meristem is epigenetically regulated

Morphogenesis, including floral meristem development, involves sequential, tissue-specific, genetically-regulated changes over time (Belmonte-Mateos and Pujades, 2022). In contrast to our broad knowledge about the spatial and genetic determinants of organ formation, much remains to be understood regarding how these factors are temporally coordinated. Across eukaryotes, temporal control of transcription during development has been associated with epigenetic factors and their interaction with transcription factors (Yosef and Regev, 2011; Kouno et al., 2013; Praggastis and Thummel, 2017; Uyehara et al., 2017; Nguyen et al., 2021). Epigenetic regulation results in dynamic and heritable changes in the chromatin environment without changes in DNA sequence. The nucleosome is the basic chromatin unit, composed of the histone octamer, containing two copies each of the histone variants H2A, H2B, H3, and H4, around which 147 bp of DNA is wrapped (Vachon et al., 2018; Baldi et al., 2020). Nucleosomes facilitate the formation of higher-order chromatin structures and enable DNA compartmentalization in the nucleus. Chromatin accessibility is a major factor influencing transcriptional activity and is regulated by chromatin remodelers and modifiers. Chromatin remodelers are classified into four main classes for yeast, animals and plants, namely the ATP-dependent chromatin remodelers switch/sucrose-non-fermenting (SWI/SNF), imitation switch (ISWI), chromodomain-helicase-DNA binding (CHD), and inositol requiring 80 (INO80), with functions in altering nucleosome position and composition (Tyagi et al., 2016; Vachon et al., 2018; Bieluszewski et al., 2023).

Chromatin modifiers configure chromatin architecture and often associate with the changes in epigenetic modification states via ‘writers’, ‘readers’, and ‘erasers’ (Allis and Jenuwein, 2016; Bieluszewski et al., 2021; Uckelmann and Davidovich, 2021). ‘Writer’ functions involve catalyzing histone or DNA modifications, while ‘reader’ functions sense chromatin states by direct association with histones, DNA, or RNA modifications or affinity for specific DNA features (Allis and Jenuwein, 2016; Uckelmann and Davidovich, 2021). Existing modifications can be removed through ‘eraser’ activity (Allis and Jenuwein, 2016; Uckelmann and Davidovich, 2021). For example, Polycomb group (PcG) proteins are transcriptional repressors that modulate histone modification to silence genes. They reside in two complexes: Polycomb repressive complex 1 (PRC1) and PRC2. PRC1 is typically associated with reader function and for further chromatin compaction, while PRC2 is well-known for its writer function by depositing repressive trimethylation of lysine 27 on histone H3 (H3K27me3) (Uckelmann and Davidovich, 2021). In plants, epigenetic regulation of transcription is associated with development and response to environmental cues, but its temporal role in driving these events is less understood (Baulcombe and Dean, 2014; Whittaker and Dean, 2017; Vachon et al., 2018). Here, we take a closer look at studies revealing the epigenetic mechanisms underlying the temporal regulation of transcription during floral meristem development. Because the floral meristem transitions from a highly proliferative meristematic state to facilitate the formation of a determinate reproductive structure, it serves as an excellent system for exploring the temporal regulation of transcription underlying developmental progression. Through these studies, we aim to demonstrate that the timing of transcriptional programs underlying developmental transitions during flower formation is epigenetically regulated.

#### 2.2.1 Flower stages 0–1: epigenetic regulation of floral meristem identity specification

**Figure 4:**
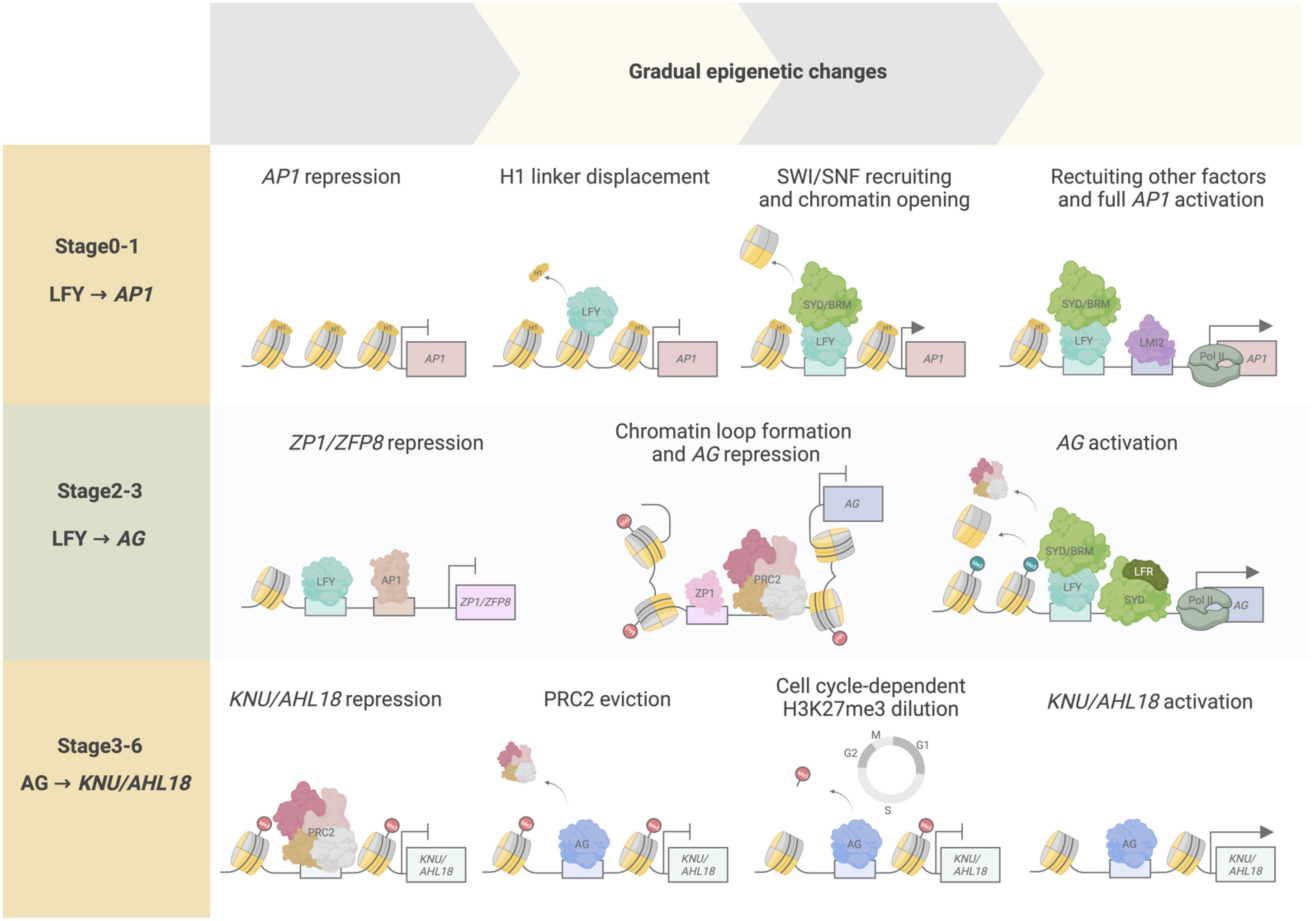
Temporal epigenetic regulation from floral stages 0 to 6. Transcription factors and chromatin regulators interact to modulate chromatin accessibility, determining transcriptional activity over time. At floral stages 0–1, LFY pioneer activity regulates *AP1* activation through initial displacement of the histone linker H1, followed by SWI/SNF chromatin recruitment and subsequent chromatin opening. These events increase chromatin accessibility, enabling other transcriptional regulators (such as LMI2 and RNA pol II) to be recruited to the *AP1* locus for full *AP1* transcriptional activation. During floral stages 2–3, LFY and AP1 act as transcriptional repressors of *ZP1* and *ZFP8* to abolish their repressive activity toward the floral homeotic genes, such as *AG*. ZP1 binds to the second intron of *AG*, a *cis*-regulatory region critical for *AG* repression by producing non-coding RNAs mediating PRC2 interaction and activity on the *AG* locus, and by facilitating *AG* chromatin loop formation. *AG* activation is accomplished by LFY-mediated SYD and BRM recruitment onto *AG* regulatory regions, acting antagonistically to PRC2 repression of the locus, and by LFR–SYD SWI/SNF complex chromatin remodeling activity promoting nucleosome repositioning or eviction and RNA poll II recruitment to *AG*. During floral stages 3–6, AG initiates floral meristem termination by activating *KNU* and *AHL18*. At stage 3, PRC2-mediated repression of *KNU* and *AHL18* is overcome by initial AG binding and PRC2 eviction from the *KNU* and *AHL18* promoter regions. H3K27me3 is passively diluted along the *KNU* and *AHL18* coding regions after approximately two cell division cycles, resulting in full *KNU* and *AHL18* activation by stage 6.

The floral meristem is a derivative of the SAM, by way of the inflorescence meristem, poised to form all floral structures upon the plant’s switch from vegetative to reproductive development. The core regulator for this specification is the helix-turn-helix transcription factor LFY (Ó’Maoiléidigh et al., 2014; Jin et al., 2021; Yamaguchi, 2021). LFY was recently formally established as a pioneer transcription factor in plants (**Figure 4**) (Jin et al., 2021). Pioneer transcription factors are distinct from conventional transcription factors as they can bind and open condensed chromatin regions, namely DNA wrapped around histones forming nucleosomes and likely in higher-order chromatin conformations. This conformation change allows the relevant transcription factors to locate and bind to the newly accessible DNA regions to activate transcription. Transcriptional activation of the floral commitment factor *APETALA1* (*AP1*) depends on the pioneer activity of LFY, which binds to the *AP1* locus in a nucleosomal state to open up local chromatin through displacement of the linker histone H1 and recruitment of SWI3B to the target site (Jin et al., 2021). SWI3B is a core component of the SPLAYED (SYD) and BRAHMA (BRM) SWITCH deficient SUCROSE NONFERMENTING (SWN/SNF) chromatin remodeling complex (Bieluszewski et al., 2023). The change in chromatin accessibility enables other transcription factors, such as LATE MERISTEM IDENTITY2 (LMI2), to bind for full activation of *AP1*. Additionally, LFY’s ability to reprogram root identity to floral meristem identity was also demonstrated to be dependent on its pioneer function (Jin et al., 2021). Previously, LFY was demonstrated to control the conversion of root explants directly into flowers, wherein the formation of rosette leaves typically preceding flower development during normal shoot development, is bypassed (Wagner et al., 2004). This suggests that LFY is sufficient for identity specification regardless of the organ (Jin et al., 2021).

LFY coordinates with the SWI2/SNF2 chromatin-remodeling ATPases SYD and BRM to orchestrate local changes in chromatin accessibility. SWI/SNF ATPase chromatin remodeling complexes are conserved across eukaryotes and regulate various nuclear events within the cell and play important functions throughout growth and development (Bieluszewski et al., 2023). SYD and BRM have both distinct and overlapping functions, especially during early flower development (Bezhani et al., 2007; Vachon et al., 2018; Pelayo et al., 2021). Recently, the comprehensive characterization of SWI/SNF complexes in plants uncovered the molecular underpinnings of the functional roles attributed to SWI/SNF remodelers such as SYD, BRM, and the newly characterized SWI/SNF ATPase subunits MINUSCULE1 [MINU1, also named CHROMATIN REMODELING12 (CHR12)] and MINU2 (also named CHR23) (Guo et al., 2022; Hernández-García et al., 2022). Specifically, SWI/SNF chromatin remodeling complexes can be classified into three main classes defined by their ATPase subunits: BRM-associated (BAS), SYD-associated (SAS), and MINU1/2-associated SWI/SNF complexes (MAS); differences in their binding affinities toward the active histone modifications histone H3 acetylation (H3ac) and H3K4me3 along their target loci likely determine the distinct and shared functions of these complexes. These results present a major advance in our understanding of the precise factors determining the distinctions and similarities attributed to SYD and BRM functions, as well as for other proposed SWI/SNF complex components. SAS complex function was specifically associated with flower development, indicating that chromatin remodeling activities during flower formation may be mediated through SAS complex activity. However, much remains to be understood in how the SAS complex, along with the other SWI/SNF classes, functions specifically in different developmental stages, such as during floral meristem identity specification, and how it coordinates its activities with pioneer transcription factors, such as LFY. Future work aimed at examining the roles of the different SWI/SNF classes in greater detail will address the current gaps in our knowledge on how the chromatin environment determines floral meristem identity.

The LFY–*AP1* activation module is also known to go through temporal delays at the onset of *AP1* induction by LFY until *AP1* is fully expressed (Wagner et al., 1999). LFY accumulates in incipient floral primordia, while the activation of *AP1* expression begins from stage 1 onward (Yamaguchi et al., 2013; Refahi et al., 2021) (**Figure 4**). LFY pioneer activity is consistent with these observed lag times and may contribute to this temporal regulation, as pioneer function is characterized by an observed residency time on target genomic sites prior to activation, leading to the initiation of chromatin remodeling that allows the binding of other factors necessary for activating the expression of target genes (Zaret and Carroll, 2011; Yamaguchi, 2021). Indeed, in root explant experiments for reprogramming to floral fate, *AP1* activation was detected only after 24 hours of *LFY* induction, with gradual accumulation of LFY protein over 5 days. Enhanced chromatin accessibility at the *AP1* locus was also observed 5 days after *LFY* induction. During floral meristem identity specification, these events may initially correspond to stages 0–2 of flower development wherein LFY and AP1 activity have been observed (Smyth et al., 1990; Ó’Maoiléidigh et al., 2014). During these stages, LFY and AP1 are also known to have downstream regulatory activities, such as promoting the expression of other floral meristem identity genes and regulating genes for phytohormone signaling for floral primordia formation, suggesting that these activities may thus be, at least in part, regulated by LFY pioneer function in activating *AP1* (Ó’Maoiléidigh et al., 2014; Goslin et al., 2017).

#### 2.2.2 Flower stages 2-3: epigenetic regulation of floral organ identity specification

Following floral meristem identity specification, LFY and AP1, together with the MADS box transcription factor SEPALATTA3 (SEP3), activate the expression of floral organ identity genes, such as *AP3* and *PISTILLATA1* (*PI*), involved in petal and stamen formation, and *AG*, involved in stamen and carpel formation, by early stage 3 (Wu et al., 2012). LFY recruits SYD and BRM to *AP3* and *AG* regulatory regions to overcome polycomb-mediated repression, resulting in *AP3* and *AG* transcription (Wu et al., 2012).

A recent study reported that LFY and AP1 mediate *AP3*, *PI*, and *AG* activation by downregulating two zinc finger protein transcription factor genes, Arabidopsis *ZINC FINGER PROTEIN1* (*ZP1*), and *ZINC FINGER PROTEIN8* (*ZFP8*) (Hu et al., 2023). ZP1 has known roles in proper root hair development, while ZP8 is associated with trichome initiation (Hu et al., 2023). Hu and colleagues showed that ZP1 and ZFP8 are transcriptional repressors of *AP3*, *PI*, and *AG* in leaves during the vegetative stage. Upon the switch to reproductive development, LFY and AP1 bind to *ZP1* and *ZFP8* promoters to repress their expression and eliminate the repression imposed by their encoded proteins on *AP3*, *PI*, and *AG* expression at stage 3 (**Figure 4**). Indeed, misexpression of *ZP1* and *ZFP8* under the control of the *LFY* or *AP1* promoter resulted in loss of floral organ identity and downregulation of *AP3*, *PI*, and *AG* expression. In a parallel approach, the *zp1 zfp8 lfy* and *zp1 zfp8 ap1* triple mutants could partially restore petal formation that is normally defective in the *lfy* and *ap1* single mutants, further supporting the observation that ZP1 and ZFP8 are involved in activating B- and C-class floral homeotic genes. ZP1 binds to regions upstream of the *AP3* and *PI* transcription start sites that are not bound by LFY, suggesting that ZP1 directly represses *AP3* and *PI* expression independently of LFY (**Figure 4**). ZP1 associates with the 3’ end of the second intron in *AG*, where LFY and the PRC2 methyltransferase CURLY LEAF (CLF) also binds, suggesting competitive binding between ZP1 and LFY for *AG* regulation (Goodrich et al., 1997; Wu et al., 2018). In addition, ZP1 may be involved in recruiting PRC2 to *AG*. However, further studies are needed to establish a link between downregulation of ZP1 and ZFP8 repressive activity and removal of PRC2-mediated repression and loss of H3K27me3 in *AG*. Whether SWI2/SNF2 complexes mediate chromatin remodeling to activate *AP3*, *PI*, and *AG* expression in coordination with ZP1 and ZFP8, and PRC2 will be interesting to study. Overall, this study reveals transcription factor interactions mediating the repression of B- and C-class floral homeotic genes.

An additional factor for *AG* activation was recently described: LEAF AND FLOWER-RELATED (LFR) is a subunit of the SAS complex and functions interdependently with SYD to activate *AG* expression (Lin et al., 2023). LFR is an Armadillo-repeat-containing nuclear protein with known roles in leaf development through its interactions with various components of the SWI2/SNF2 chromatin complex, such as SWI3B and SWIP37B (Vercruyssen et al., 2014; Nelissen et al., 2015; Lin et al., 2018; Lin et al., 2021). Lin and colleagues found additional SWI2/SNF2 subunits that interact with LFR, namely SYD, SWI3A, SWI3B, and ACTIN-RELATED PROTEIN4 (ARP4). Furthermore, the N-terminal region of SYD, which contains its QLQ domain for establishing protein-protein interactions, was necessary and sufficient to establish the physical interaction with LFR (Sang et al., 2012). Phenotypic and expression analyses of the *syd lfr* double mutant suggested that SYD and LFR share functions in establishing proper stamen and pistil development through the activation of *AG* expression. LFR directly binds to regions upstream of the *AG* transcription start site and to the *AG* second intron, a region previously shown to be directly bound by SYD (**Figure 4**) (Wu et al., 2012). The *AG* locus forms a chromatin loop that directly affects its transcriptional regulation (Wu et al., 2018). SYD and LFR inhibit chromatin loop formation and PRC2 accumulation, while promoting nucleosome sliding or eviction and recruitment of the RNA polymerase II complex to the *AG* locus to activate its transcription (**Figure 4**). These results suggest that SAS complex activity with the LFR variant is specifically involved in *AG* activation during stage 3 of early flower development and is functionally important for proper stamen and carpel development.

#### 2.2.3 Flower stages 3-6: epigenetic regulation of floral meristem determinacy and floral organ development

Termination of the floral meristem is initiated at stage 6 of early flower development by the C-class homeotic protein AG. Floral meristem termination is irreversible and commit floral meristem cells to a determinate fate. To achieve this termination, AG directly and indirectly represses the expression of the homeodomain transcription factor gene *WUSCHEL* (*WUS*); WUS function sustains stem cell production in the floral meristem (Mayer et al., 1998; Liu et al., 2011; Xu et al., 2019; Min and Kramer, 2023). The indirect repression of *WUS* by AG involves distinct but parallel pathways with the C2H2-type zinc finger transcription factor KNUCKLES (KNU) and the YABBY transcription factor CRABS CLAW (CRC) (Sun et al., 2009; Yamaguchi et al., 2017; Yamaguchi et al., 2018). AG activates *KNU* for floral meristem termination through cell cycle–dependent dilution of H3K27me3 at the *KNU* locus, leading to time-delayed *KNU* activation. Specifically, by stage 2 of flower development, *KNU* expression is repressed by PRC2 bound to the *KNU* locus, which establishes and maintains H3K27me3 marks along its coding sequence (**Figure 4**) (Sun et al., 2009; Sun et al., 2014; Ikeuchi et al., 2015). At floral stage 3, AG accumulation leads to PRC2 eviction at the *KNU* promoter region followed by a decrease in PRC2-mediated H3K27me3 deposition along the *KNU* coding region, which occurs in a cell cycle–dependent manner (**Figure 4**). As a result, *KNU* expression is activated after two days of cell divisions just before floral meristem termination at floral stage 6 (**Figure 4**) (Sun et al., 2014). Altering *KNU* temporal expression results in either premature floral meristem termination or in its indeterminacy (Sun et al., 2009). Hence, *KNU* was the first gene described as being transcriptionally activated via cell cycle–dependent epigenetic regulation by AG.

A recent study identified additional targets that are regulated by AG in a similar manner. It also delved into the mechanistic determinants of this intrinsic timing mechanism (‘biotimer’) through genome-wide approaches (Pelayo et al., 2023). Additional AG biotimer targets were identified based on four main criteria: 1) direct AG binding to target genes, together with 2) PRC2 eviction at these sites resulting in 3) cell cycle–dependent H3K27me3 dilution from stages 3 to 5, and 4) subsequent transcriptional activation from stages 5 to 6. Through this screening, *KNU* was among the candidate AG-mediated biotimer targets, as well as *AT HOOK MOTIF NUCLEAR LOCALIZED PROTEIN18* (*AHL18*) and *PLATZ10*. *AHL18* encodes a known regulator of lateral root development, while *PLATZ10* encodes a transcription factor that belongs to a family of plant-specific zinc-dependent DNA-binding proteins (Nagano et al., 2001; Širl et al., 2020). A mathematical model quantitatively generalizing the biotimer mechanism was also introduced, wherein the timing of transcriptional activation was correlated with the length of H3K27me3-marked regions in biotimer genes. The time of induction after the onset of AG binding for *KNU*, *AHL18*, and *PLATZ10* was approximately 1.7–2.52, 2.45–4.90, and 3.73–7.46 days, respectively, based on the predictions made using the model. The corresponding floral stage–specific expression would be stages 5–6 for *KNU*, stages 5–7 for *AHL18*, and stages 6–9 for *PLATZ10*. These stages are consistent with the observed tissue-specific expression pattern for *KNU*, *AHL18*, and *PLATZ10* as well as with the associated functions in stamen development for *AHL18* and *PLATZ10*. *AHL18* is expressed in nascent stamen filament tissue from floral stages 5 to 8, while *PLATZ10* is expressed in anthers from stage 8 floral buds. Stamen primordia giving rise to stamen filaments occur from stage 7, in accordance with the predicted and observed *AHL18* spatiotemporal expression pattern (**Figure 4**). Similarly, anther locule formation initiates at stage 8 and is consistent with the predicted and observed *PLATZ10* spatiotemporal expression pattern. These results suggest that the biotimer mechanism is prevalent throughout flower development and contributes to transcriptional regulation, not only for floral meristem termination but also for proper floral organ development. In addition, the current set of criteria defining the biotimer mechanism are sufficient parameters to quantifiably determine biotimer behavior and to make accurate predictions through this model.

Experimental validation of the model’s predictions was carried out by modifying the number of *del* repeats in the *KNU* locus. *del* is an H3K27me3-dense and FERTILIZATION INDEPENDENT ENDOSPERM (FIE)-bound region previously identified as being necessary for proper timing of *KNU* transcriptional activation by deletion of this region (hence, the name ‘*del*’) (Sun et al., 2009). Iterative addition of *del* copies delayed and decreased *KNU* expression in line with the predictions for temporal transcriptional activation. Further characterization with *del* mutants also showed that transcriptional activation was modulated in a PRC2- and cell cycle–dependent manner. Taken together, this work highlighted an example of temporal transcriptional regulation in an AG-directed manner that is modulated by a dynamic interplay between PRC2 activity, chromatin environment, and the cell cycle.

KNU mediates floral meristem termination by directly repressing *WUS*, but the extent of the role played by KNU in fully ceasing cell proliferation in the floral meristem was unknown. Shang et al. (2021) examined the range of KNU repressive transcriptional activities in the floral meristem to elucidate floral termination control within a limited temporal context (between floral stages 6 to 8). Floral meristem proliferation activity ceases by floral stage 6 when *WUS* is silenced by KNU (Shang et al., 2019). KNU forms a part of two different repressive complexes to disrupt WUS-mediated meristem maintenance. One complex is a histone deacetylase (HDA) complex composed of the co-repressor TOPLESS (TPL), HDA19, and the protein encoded by the AG target gene *MINI ZINC FINGER2* (*MIF2*), which acts as an adaptor protein between KNU, TPL, and HDA19 (Bollier et al., 2018; Pelayo et al., 2021). The other repressive complex is a KNU–PRC2 complex. Initially, KNU binding to the *WUS* locus results in eviction of the *WUS* activator SYD and subsequent chromatin compaction. KNU then mediates PRC2 binding to the *WUS* locus by interacting with the PRC2 component FIE, and stable *WUS* repression is achieved by deposition of H3K27me3 marks on the gene region (Sun et al., 2019; Pelayo et al., 2021). In this study, the authors demonstrated that KNU activity for floral meristem termination is not limited to *WUS* repression but also includes the direct repression of other components of the WUS feedback loop for meristem maintenance, namely the WUS target *CLAVATA3* (*CLV3*) and *CLV1*, encoding the CLV3 polypeptide and its cognate receptor CLV1. *CLV3* and *CLV1* expression decreases upon *KNU* induction; in addition, KNU binds directly to the *CLV3* and *CLV1* promoters, indicating that KNU directly represses both genes. Additionally, significant H3K27me3 enrichment at the *CLV3* locus may also be a consequence of KNU binding through establishing the KNU–PRC2 complex on the locus. *In vitro*, KNU was shown to disrupt WUS–WUS and WUS–HAIRY MERISTEM1 (HAM1) interactions essential for WUS-mediated meristem maintenance. The authors also showed that KNU functions across all cell layers of the floral meristem for proper flower formation. Multilevel KNU repressive activity in the floral meristem coordinated by transcription factor networks and dynamic histone regulation result in a robust regulatory module for floral meristem termination ensuring that meristem maintenance activities will cease within the appropriate spatiotemporal context for proper flower development.

### 2.3 Conclusions and future prospects

The floral meristem is a pivotal aspect of floral architecture as it gives rise to all floral structures. Floral meristem development, including floral meristem specification and determinacy, takes place in a 4D space, as it is regulated in 3D and over time. Studying floral meristem regulation in four dimensions can be challenging, as most available techniques can only capture one or two dimensions of this progression at a time. However, recent technical and conceptual advances now allow the examination of flower development, particularly within the floral meristem, in four dimensions. This close examination is achieved through spatially reconstructed 3D atlases that integrate transcriptional inputs with morphological outcomes in real time, as well as through high-resolution transcription factor–chromatin interactions, which have a substantial influence on temporal transcriptional regulation across developmental transitions. Future work directed at integrating transcriptomic data at single-cell resolution with the epigenome, which was recently reported for mammalian systems but currently unavailable in plants, should greatly advance our understanding of floral meristem development in four dimensions (Minton, 2023; Zhang et al., 2023). For example, the use of these atlases to examine epigenetic factor expression patterns and their downstream targets within the context of their different functions at distinct floral stages will be of significant interest. In addition, although there is substantial knowledge about the transcriptional control underlying floral meristem development, much remains to be understood regarding the genetic and epigenetic mechanisms behind this regulation. For example, how the floral homeotic transcription factors achieve regulatory specificity remains unclear; how chromatin remodelers and modifiers exert their tissue-specific activities is only recently being uncovered (Bieluszewski et al., 2021; Bieluszewski et al., 2023; Goslin et al., 2023). Addressing these issues will be vital in shaping a holistic view of floral meristem development, as well as of overall flower development.

## Author contributions

MP: Conceptualization, Writing – original draft, Writing – review & editing. NY: Conceptualization, Funding acquisition, Writing – original draft, Writing – review & editing.

## References

[B1] Allis C. D. Jenuwein T. (2016). The molecular hallmarks of epigenetic control. Nat. Rev. Genet. 17, 487–500. doi: 10.1038/nrg.2016.59 27346641

[B2] Alvarez-Buylla E. R. Benítez M. Corvera-Poiré A. Cador Á.C. Folter S. de Buen A. G.. (2010). Flower development. arbo.j 2010. doi: 10.1199/tab.0127

[B3] Baldi S. Korber P. Becker P. B. (2020). Beads on a string—nucleosome array arrangements and folding of the chromatin fiber. Nat. Struct. Mol. Biol. 27, 109–118. doi: 10.1038/s41594-019-0368-x 32042149

[B4] Bassel G. W. Smith R. S. (2016). Quantifying morphogenesis in plants in 4D. Curr. Opin. Plant Biol. 29, 87–94. doi: 10.1016/j.pbi.2015.11.005 26748353

[B5] Baulcombe D. C. Dean C. (2014). Epigenetic regulation in plant responses to the environment. Cold Spring Harb. Perspect. Biol. 6, a019471. doi: 10.1101/cshperspect.a019471 25183832 PMC4142964

[B6] Belmonte-Mateos C. Pujades C. (2022). From cell states to cell fates: how cell proliferation and neuronal differentiation are coordinated during embryonic development. Front. Neurosci. 15. doi: 10.3389/fnins.2021.781160 PMC8761814 35046768

[B7] Besnard F. Rozier F. Vernoux T. (2014). The AHP6 cytokinin signaling inhibitor mediates an auxin-cytokinin crosstalk that regulates the timing of organ initiation at the shoot apical meristem. Plant Signal Behav. 9, e28788. doi: 10.4161/psb.28788 24732036 PMC4091322

[B8] Bezhani S. Winter C. Hershman S. Wagner J. D. Kennedy J. F. Kwon C. S.. (2007). Unique, shared, and redundant roles for the arabidopsis SWI/SNF chromatin remodeling ATPases BRAHMA and SPLAYED. Plant Cell 19, 403–416. doi: 10.1105/tpc.106.048272 17293567 PMC1867337

[B9] Bieluszewski T. Prakash S. Roulé T. Wagner D. (2023). The role and activity of SWI/SNF chromatin remodelers. Annu. Rev. Plant Biol. 74, 139–163. doi: 10.1146/annurev-arplant-102820-093218 36889009

[B10] Bieluszewski T. Xiao J. Yang Y. Wagner D. (2021). PRC2 activity, recruitment, and silencing: a comparative perspective. Trends Plant Sci. 26, 1186–1198. doi: 10.1016/j.tplants.2021.06.006 34294542

[B11] Bollier N. Sicard A. Leblond J. Latrasse D. Gonzalez N. Gévaudant F.. (2018). At-MINI ZINC FINGER2 and sl-INHIBITOR OF MERISTEM ACTIVITY, a conserved missing link in the regulation of floral meristem termination in arabidopsis and tomato. Plant Cell 30, 83–100. doi: 10.1105/tpc.17.00653 29298836 PMC5810569

[B12] Bowman J. L. Sakai H. Jack T. Weigel D. Mayer U. Meyerowitz E. M. (1992). Superman, a regulator of floral homeotic genes in Arabidopsis. Development 114, 599–615. doi: 10.1242/dev.114.3.599 1352237

[B13] Bowman J. L. Smyth D. R. Meyerowitz E. M. (1989). Genes directing flower development in Arabidopsis. Plant Cell 1, 37–52. doi: 10.1105/tpc.1.1.37 2535466 PMC159735

[B14] Bowman J. L. Smyth D. R. Meyerowitz E. M. (1991). Genetic interactions among floral homeotic genes of Arabidopsis. Development 112, 1–20. doi: 10.1242/dev.112.1.1 1685111

[B15] Bowman J. L. Smyth D. R. Meyerowitz E. M. (2012). The ABC model of flower development: then and now. Development 139, 4095–4098. doi: 10.1242/dev.083972 23093420

[B16] Brand U. Fletcher J. C. Hobe M. Meyerowitz E. M. Simon R. (2000). Dependence of stem cell fate in arabidopsis on a feedback loop regulated by CLV3 activity. Science 289, 617–619. doi: 10.1126/science.289.5479.617 10915624

[B17] Brunoud G. Wells D. M. Oliva M. Larrieu A. Mirabet V. Burrow A. H.. (2012). A novel sensor to map auxin response and distribution at high spatio-temporal resolution. Nature 482, 103–106. doi: 10.1038/nature10791 22246322

[B18] Casson S. Spencer M. Walker K. Lindsey K. (2005). Laser capture microdissection for the analysis of gene expression during embryogenesis of Arabidopsis. Plant J. 42, 111–123. doi: 10.1111/j.1365-313X.2005.02355.x 15773857

[B19] Chen C. Ge Y. Lu L. (2023). Opportunities and challenges in the application of single-cell and spatial transcriptomics in plants. Front. Plant Sci. 14. doi: 10.3389/fpls.2023.1185377 PMC10453814 37636094

[B20] Chen D. Yan W. Fu L.-Y. Kaufmann K. (2018). Architecture of gene regulatory networks controlling flower development in Arabidopsis thaliana. Nat. Commun. 9, 4534. doi: 10.1038/s41467-018-06772-3 30382087 PMC6208445

[B21] Chen Q. Atkinson A. Otsuga D. Christensen T. Reynolds L. Drews G. N. (1999). The Arabidopsis FILAMENTOUS FLOWER gene is required for flower formation. Development 126, 2715–2726. doi: 10.1242/dev.126.12.2715 10331982

[B22] Coen E. S. Meyerowitz E. M. (1991). The war of the whorls: genetic interactions controlling flower development. Nature 353, 31–37. doi: 10.1038/353031a0 1715520

[B23] Daum G. Medzihradszky A. Suzaki T. Lohmann J. U. (2014). A mechanistic framework for noncell autonomous stem cell induction in Arabidopsis. Proc. Natl. Acad. Sci. 111, 14619–14624. doi: 10.1073/pnas.1406446111 25246576 PMC4210042

[B24] Deal R. B. Henikoff S. (2011). The INTACT method for cell type-specific gene expression and chromatin profiling in Arabidopsis. Nat. Protoc. 6, 56–68. doi: 10.1038/nprot.2010.175 21212783 PMC7219316

[B25] Dennis L. Peacock J. (2019). Genes directing flower development in arabidopsis. Plant Cell 31, 1192–1193. doi: 10.1105/tpc.19.00276 31036593 PMC6588311

[B26] Drews G. N. Bowman J. L. Meyerowitz E. M. (1991). Negative regulation of the Arabidopsis homeotic gene AGAMOUS by the APETALA2 product. Cell 65, 991–1002. doi: 10.1016/0092-8674(91)90551-9 1675158

[B27] Endress P. K. (2006). “Angiosperm floral evolution: morphological developmental framework,” in Advances in botanical research developmental genetics of the flower (California, USA: Academic Press), 1–61. doi: 10.1016/S0065-2296(06)44001-5

[B28] Engelhorn J. Wellmer F. Carles C. C. (2018). “Profiling histone modifications in synchronized floral tissues for quantitative resolution of chromatin and transcriptome dynamics,” in Plant chromatin dynamics: methods and protocols methods in molecular biology. Eds. Bemer M. Baroux C. (New York, NY: Springer), 271–296. doi: 10.1007/978-1-4939-7318-7_16 29052197

[B29] Galbraith D. W. (2014). “Flow cytometry and sorting in arabidopsis,” in Arabidopsis protocols methods in molecular biology. Eds. Sanchez-Serrano J. J. Salinas J. (Totowa, NJ: Humana Press), 509–537. doi: 10.1007/978-1-62703-580-4_27 24057384

[B30] Giacomello S. Salmén F. Terebieniec B. K. Vickovic S. Navarro J. F. Alexeyenko A.. (2017). Spatially resolved transcriptome profiling in model plant species. Nat. Plants 3, 1–11. doi: 10.1038/nplants.2017.61 28481330

[B31] Godin C. Golé C. Douady S. (2020). Phyllotaxis as geometric canalization during plant development. Development 147, dev165878. doi: 10.1242/dev.165878 33046454

[B32] Goodrich J. Puangsomlee P. Martin M. Long D. Meyerowitz E. M. Coupland G. (1997). A Polycomb-group gene regulates homeotic gene expression in Arabidopsis. Nature 386, 44–51. doi: 10.1038/386044a0 9052779

[B33] Goslin K. Finocchio A. Wellmer F. (2023). Floral homeotic factors: A question of specificity. Plants (Basel) 12, 1128. doi: 10.3390/plants12051128 36903987 PMC10004826

[B34] Goslin K. Zheng B. Serrano-Mislata A. Rae L. Ryan P. T. Kwaśniewska K.. (2017). Transcription factor interplay between LEAFY and APETALA1/CAULIFLOWER during floral initiation1. Plant Physiol. 174, 1097–1109. doi: 10.1104/pp.17.00098 28385730 PMC5462026

[B35] Guo J. Cai G. Li Y.-Q. Zhang Y.-X. Su Y.-N. Yuan D.-Y.. (2022). Comprehensive characterization of three classes of Arabidopsis SWI/SNF chromatin remodelling complexes. Nat. Plants 8, 1423–1439. doi: 10.1038/s41477-022-01282-z 36471048

[B36] Heisler M. G. Ohno C. Das P. Sieber P. Reddy G. V. Long J. A.. (2005). Patterns of auxin transport and gene expression during primordium development revealed by live imaging of the arabidopsis inflorescence meristem. Curr. Biol. 15, 1899–1911. doi: 10.1016/j.cub.2005.09.052 16271866

[B37] Hernández-García J. Diego-Martin B. Kuo P. H. Jami-Alahmadi Y. Vashisht A. A. Wohlschlegel J.. (2022). Comprehensive identification of SWI/SNF complex subunits underpins deep eukaryotic ancestry and reveals new plant components. Commun. Biol. 5, 1–11. doi: 10.1038/s42003-022-03490-x 35668117 PMC9170682

[B38] Hölscher D. Schneider B. (2008). “Application of laser-assisted microdissection for tissue and cell-specific analysis of RNA, proteins, and metabolites,” in Progress in botany progress in botany. Eds. Lüttge U. Beyschlag W. Murata J. (Berlin, Heidelberg: Springer), 141–167. doi: 10.1007/978-3-540-72954-9_6

[B39] Houben A. Orford S. J. Timmis J. N. (2006). “ *In situ* hybridization to plant tissues and chromosomes,” in In situ hybridization protocols methods in molecular biology^TM^ . Eds. Darby I. A. Hewitson T. D. (Totowa, NJ: Humana Press), 203–218. doi: 10.1385/1-59745-007-3:203 16780203

[B40] Hu T. Li X. Du L. Manuela D. Xu M. (2023). LEAFY and APETALA1 down-regulate ZINC FINGER PROTEIN 1 and 8 to release their repression on class B and C floral homeotic genes. Proc. Natl. Acad. Sci. 120, e2221181120. doi: 10.1073/pnas.2221181120 37216511 PMC10235997

[B41] Ikeuchi M. Iwase A. Sugimoto K. (2015). Control of plant cell differentiation by histone modification and DNA methylation. Curr. Opin. Plant Biol. 28, 60–67. doi: 10.1016/j.pbi.2015.09.004 26454697

[B42] Irish V. (2017). The ABC model of floral development. Curr. Biol. 27, R887–R890. doi: 10.1016/j.cub.2017.03.045 28898659

[B43] Jensen E. (2014). Technical review: *in situ* hybridization. Anatomical Rec. 297, 1349–1353. doi: 10.1002/ar.22944 24810158

[B44] Jin R. Klasfeld S. Zhu Y. Fernandez Garcia M. Xiao J. Han S.-K.. (2021). LEAFY is a pioneer transcription factor and licenses cell reprogramming to floral fate. Nat. Commun. 12, 626. doi: 10.1038/s41467-020-20883-w 33504790 PMC7840934

[B45] Klepikova A. V. Kasianov A. S. Gerasimov E. S. Logacheva M. D. Penin A. A. (2016). A high resolution map of the Arabidopsis thaliana developmental transcriptome based on RNA-seq profiling. Plant J. 88, 1058–1070. doi: 10.1111/tpj.13312 27549386

[B46] Klepikova A. V. Kulakovskiy I. V. Kasianov A. S. Logacheva M. D. Penin A. A. (2019). An update to database TraVA: organ-specific cold stress response in Arabidopsis thaliana. BMC Plant Biol. 19, 49. doi: 10.1186/s12870-019-1636-y 30813912 PMC6393959

[B47] Kouno T. de Hoon M. Mar J. C. Tomaru Y. Kawano M. Carninci P.. (2013). Temporal dynamics and transcriptional control using single-cell gene expression analysis. Genome Biol. 14, R118. doi: 10.1186/gb-2013-14-10-r118 24156252 PMC4015031

[B48] Kwiatkowska D. (2006). Flower primordium formation at the Arabidopsis shoot apex: quantitative analysis of surface geometry and growth. J. Exp. Bot. 57, 571–580. doi: 10.1093/jxb/erj042 16377735

[B49] Kwiatkowska D. (2008). Flowering and apical meristem growth dynamics. J. Exp. Bot. 59, 187–201. doi: 10.1093/jxb/erm290 18256052

[B50] Kwon C. S. Hibara K. Pfluger J. Bezhani S. Metha H. Aida M.. (2006). A role for chromatin remodeling in regulation of CUC gene expression in the Arabidopsis cotyledon boundary. Development 133, 3223–3230. doi: 10.1242/dev.02508 16854978

[B51] Lenhard M. Bohnert A. Jürgens G. Laux T. (2001). Termination of stem cell maintenance in arabidopsis floral meristems by interactions between WUSCHEL and AGAMOUS. Cell 105, 805–814. doi: 10.1016/S0092-8674(01)00390-7 11440722

[B52] Lin X. Gu D. Zhao H. Peng Y. Zhang G. Yuan T.. (2018). LFR is functionally associated with AS2 to mediate leaf development in Arabidopsis. Plant J. 95, 598–612. doi: 10.1111/tpj.13973 29775508

[B53] Lin X. Yuan T. Guo H. Guo Y. Yamaguchi N. Wang S.. (2023). The regulation of chromatin configuration at AGAMOUS locus by LFR-SYD-containing complex is critical for reproductive organ development in Arabidopsis. Plant J. 478–496. doi: 10.1111/tpj.16385 37478313

[B54] Lin X. Yuan C. Zhu B. Yuan T. Li X. Yuan S.. (2021). LFR physically and genetically interacts with SWI/SNF component SWI3B to regulate leaf blade development in arabidopsis. Front. Plant Sci. 12. doi: 10.3389/fpls.2021.717649 PMC8385146 34456957

[B55] Liu X. Kim Y. J. Müller R. Yumul R. E. Liu C. Pan Y.. (2011). AGAMOUS terminates floral stem cell maintenance in arabidopsis by directly repressing WUSCHEL through recruitment of polycomb group proteins. Plant Cell 23, 3654–3670. doi: 10.1105/tpc.111.091538 22028461 PMC3229141

[B56] Marx V. (2021). Method of the Year: spatially resolved transcriptomics. Nat. Methods 18, 9–14. doi: 10.1038/s41592-020-01033-y 33408395

[B57] Mayer K. F. X. Schoof H. Haecker A. Lenhard M. Jürgens G. Laux T. (1998). Role of WUSCHEL in regulating stem cell fate in the arabidopsis shoot meristem. Cell 95, 805–815. doi: 10.1016/S0092-8674(00)81703-1 9865698

[B58] Min Y. Kramer E. M. (2023). All’s well that ends well: the timing of floral meristem termination. New Phytol. 238, 500–505. doi: 10.1111/nph.18715 36600362

[B59] Minton K. (2023). Layering epigenomic and transcriptomic space. Nat. Rev. Genet. 24, 273–273. doi: 10.1038/s41576-023-00596-8 36973496

[B60] Moriel N. Senel E. Friedman N. Rajewsky N. Karaiskos N. Nitzan M. (2021). NovoSpaRc: flexible spatial reconstruction of single-cell gene expression with optimal transport. Nat. Protoc. 16, 4177–4200. doi: 10.1038/s41596-021-00573-7 34349282

[B61] Nagano Y. Furuhashi H. Inaba T. Sasaki Y. (2001). A novel class of plant-specific zinc-dependent DNA-binding protein that binds to A/T-rich DNA sequences. Nucleic Acids Res. 29, 4097–4105. doi: 10.1093/nar/29.20.4097 11600698 PMC60209

[B62] Nelissen H. Eeckhout D. Demuynck K. Persiau G. Walton A. van Bel M.. (2015). Dynamic changes in ANGUSTIFOLIA3 complex composition reveal a growth regulatory mechanism in the maize leaf. Plant Cell 27, 1605–1619. doi: 10.1105/tpc.15.00269 26036253 PMC4498210

[B63] Neumann M. Xu X. Smaczniak C. Schumacher J. Yan W. Blüthgen N.. (2022). A 3D gene expression atlas of the floral meristem based on spatial reconstruction of single nucleus RNA sequencing data. Nat. Commun. 13, 2838. doi: 10.1038/s41467-022-30177-y 35595749 PMC9122980

[B64] Nguyen P. Pease N. A. Kueh H. Y. (2021). Scalable control of developmental timetables by epigenetic switching networks. J. R. Soc. Interface 18, 20210109. doi: 10.1098/rsif.2021.0109 34283940 PMC8292019

[B65] Nitzan M. Karaiskos N. Friedman N. Rajewsky N. (2019). Gene expression cartography. Nature 576, 132–137. doi: 10.1038/s41586-019-1773-3 31748748

[B66] Nobori T. Oliva M. Lister R. Ecker J. R. (2023). Multiplexed single-cell 3D spatial gene expression analysis in plant tissue using PHYTOMap. Nat. Plants 9, 1026–1033. doi: 10.1038/s41477-023-01439-4 37308583 PMC10356616

[B67] Ó’Maoiléidigh D. S. Graciet E. Wellmer F. (2014). Gene networks controlling Arabidopsis thaliana flower development. New Phytol. 201, 16–30. doi: 10.1111/nph.12444 23952532

[B68] Pajoro A. Madrigal P. Muiño J. M. Matus J. T. Jin J. Mecchia M. A.. (2014). Dynamics of chromatin accessibility and gene regulation by MADS-domain transcription factors in flower development. Genome Biol. 15, R41. doi: 10.1186/gb-2014-15-3-r41 24581456 PMC4054849

[B69] Parcy F. Nilsson O. Busch M. A. Lee I. Weigel D. (1998). A genetic framework for floral patterning. Nature 395, 561–566. doi: 10.1038/26903 9783581

[B70] Peiffer J. A. Kaushik S. Sakai H. Arteaga-Vazquez M. Sanchez-Leon N. Ghazal H.. (2008). A spatial dissection of the Arabidopsis floral transcriptome by MPSS. BMC Plant Biol. 8, 43. doi: 10.1186/1471-2229-8-43 18426585 PMC2375892

[B71] Pelayo M. A. Morishita F. Sawada H. Matsushita K. Iimura H. He Z.. (2023). AGAMOUS regulates various target genes *via* cell cycle–coupled H3K27me3 dilution in floral meristems and stamens. Plant Cell 35, 2821–2847. doi: 10.1093/plcell/koad123 37144857 PMC10396370

[B72] Pelayo M. A. Yamaguchi N. Ito T. (2021). One factor, many systems: the floral homeotic protein AGAMOUS and its epigenetic regulatory mechanisms. Curr. Opin. Plant Biol. 61, 102009. doi: 10.1016/j.pbi.2021.102009 33640614

[B73] Petrášek J. Friml J. (2009). Auxin transport routes in plant development. Development 136, 2675–2688. doi: 10.1242/dev.030353 19633168

[B74] Praggastis S. A. Thummel C. S. (2017). Right time, right place: the temporal regulation of developmental gene expression. Genes Dev. 31, 847–848. doi: 10.1101/gad.301002.117 28566535 PMC5458752

[B75] Reddy G. V. Heisler M. G. Ehrhardt D. W. Meyerowitz E. M. (2004). Real-time lineage analysis reveals oriented cell divisions associated with morphogenesis at the shoot apex of Arabidopsis thaliana. Development 131, 4225–4237. doi: 10.1242/dev.01261 15280208

[B76] Refahi Y. Zardilis A. Michelin G. Wightman R. Leggio B. Legrand J.. (2021). A multiscale analysis of early flower development in Arabidopsis provides an integrated view of molecular regulation and growth control. Dev. Cell 56, 540–556.e8. doi: 10.1016/j.devcel.2021.01.019 33621494 PMC8519405

[B77] Reinhardt D. Pesce E.-R. Stieger P. Mandel T. Baltensperger K. Bennett M.. (2003). Regulation of phyllotaxis by polar auxin transport. Nature 426, 255–260. doi: 10.1038/nature02081 14628043

[B78] Sang Y. Silva-Ortega C. O. Wu S. Yamaguchi N. Wu M.-F. Pfluger J.. (2012). Mutations in two non-canonical Arabidopsis SWI2/SNF2 chromatin remodeling ATPases cause embryogenesis and stem cell maintenance defects. Plant J. 72, 1000–1014. doi: 10.1111/tpj.12009 23062007 PMC3561502

[B79] Sauquet H. von Balthazar M. Magallón S. Doyle J. A. Endress P. K. Bailes E. J.. (2017). The ancestral flower of angiosperms and its early diversification. Nat. Commun. 8, 16047. doi: 10.1038/ncomms16047 28763051 PMC5543309

[B80] Scheres B. (1998). A LEAFY link from outer space. Nature 395, 545–547. doi: 10.1038/26858 9783577

[B81] Schmid M. Davison T. S. Henz S. R. Pape U. J. Demar M. Vingron M.. (2005). A gene expression map of Arabidopsis thaliana development. Nat. Genet. 37, 501–506. doi: 10.1038/ng1543 15806101

[B82] Shang E. Ito T. Sun B. (2019). Control of floral stem cell activity in Arabidopsis. Plant Signal Behav. 14, 1659706. doi: 10.1080/15592324.2019.1659706 31462133 PMC6804719

[B83] Shang E. Wang X. Li T. Guo F. Ito T. Sun B.. (2021). Robust control of floral meristem determinacy by position-specific multifunctions of KNUCKLES. Proceedings of the National Academy of Sciences 118, e2102826118. doi: 10.1073/pnas.2102826118 PMC8433499 34462349

[B84] Shi D. Jouannet V. Agustí J. Kaul V. Levitsky V. Sanchez P.. (2021). Tissue-specific transcriptome profiling of the Arabidopsis inflorescence stem reveals local cellular signatures. Plant Cell 33, 200–223. doi: 10.1093/plcell/koaa019 33582756 PMC8136906

[B85] Širl M. Šnajdrová T. Gutiérrez-Alanís D. Dubrovsky J. G. Vielle-Calzada J. P. Kulich I.. (2020). At-hook motif nuclear localised protein 18 as a novel modulator of root system architecture. Int. J. Mol. Sci. 21, 1886. doi: 10.3390/ijms21051886 32164240 PMC7084884

[B86] Smaczniak C. Immink R. G. H. Muiño J. M. Blanvillain R. Busscher M. Busscher-Lange J.. (2012). Characterization of MADS-domain transcription factor complexes in *Arabidopsis* flower development. Proc. Natl. Acad. Sci. U.S.A. 109, 1560–1565. doi: 10.1073/pnas.1112871109 22238427 PMC3277181

[B87] Smyth D. R. Bowman J. L. Meyerowitz E. M. (1990). Early flower development in Arabidopsis. Plant Cell 2, 755–767. doi: 10.1105/tpc.2.8.755 2152125 PMC159928

[B88] Ståhl P. L. Salmén F. Vickovic S. Lundmark A. Navarro J. F. Magnusson J.. (2016). Visualization and analysis of gene expression in tissue sections by spatial transcriptomics. Science 353, 78–82. doi: 10.1126/science.aaf2403 27365449

[B89] Sun B. Looi L.-S. Guo S. He Z. Gan E.-S. Huang J.. (2014). Timing mechanism dependent on cell division is invoked by polycomb eviction in plant stem cells. Science 343, 1248559. doi: 10.1126/science.1248559 24482483

[B90] Sun B. Xu Y. Ng K.-H. Ito T. (2009). A timing mechanism for stem cell maintenance and differentiation in the Arabidopsis floral meristem. Genes Dev. 23, 1791–1804. doi: 10.1101/gad.1800409 19651987 PMC2720260

[B91] Sun B. Zhou Y. Cai J. Shang E. Yamaguchi N. Xiao J.. (2019). Integration of transcriptional repression and polycomb-mediated silencing of WUSCHEL in floral meristems. Plant Cell 31, 1488–1505. doi: 10.1105/tpc.18.00450 31068455 PMC6635863

[B92] Takada S. Hibara K. Ishida T. Tasaka M. (2001). The CUP-SHAPED COTYLEDON1 gene of Arabidopsis regulates shoot apical meristem formation. Development 128, 1127–1135. doi: 10.1242/dev.128.7.1127 11245578

[B93] Talbert P. B. Adler H. T. Parks D. W. Comai L. (1995). The REVOLUTA gene is necessary for apical meristem development and for limiting cell divisions in the leaves and stems of Arabidopsis thaliana. Development 121, 2723–2735. doi: 10.1242/dev.121.9.2723 7555701

[B94] Thomson B. Zheng B. Wellmer F. (2017). Floral organogenesis: when knowing your ABCs is not enough1[OPEN]. Plant Physiol. 173, 56–64. doi: 10.1104/pp.16.01288 27789738 PMC5210729

[B95] Tyagi M. Imam N. Verma K. Patel A. K. (2016). Chromatin remodelers: We are the drivers Nucleus 7, 388–404. doi: 10.1080/19491034.2016.1211217 27429206 PMC5039004

[B96] Uckelmann M. Davidovich C. (2021). Not just a writer: PRC2 as a chromatin reader. Biochem. Soc. Trans. 49, 1159–1170. doi: 10.1042/BST20200728 34060617 PMC8286813

[B97] Uyehara C. M. Nystrom S. L. Niederhuber M. J. Leatham-Jensen M. Ma Y. Buttitta L. A.. (2017). Hormone-dependent control of developmental timing through regulation of chromatin accessibility. Genes Dev. 31, 862–875. doi: 10.1101/gad.298182.117 28536147 PMC5458754

[B98] Vachon G. Engelhorn J. Carles C. C. (2018). Interactions between transcription factors and chromatin regulators in the control of flower development. J. Exp. Bot. 69, 2461–2471. doi: 10.1093/jxb/ery079 29506187

[B99] Vanneste S. Friml J. (2009). Auxin: A trigger for change in plant development. Cell 136, 1005–1016. doi: 10.1016/j.cell.2009.03.001 19303845

[B100] Vercruyssen L. Verkest A. Gonzalez N. Heyndrickx K. S. Eeckhout D. Han S.-K.. (2014). ANGUSTIFOLIA3 binds to SWI/SNF chromatin remodeling complexes to regulate transcription during arabidopsis leaf development. Plant Cell 26, 210–229. doi: 10.1105/tpc.113.115907 24443518 PMC3963571

[B101] Vijayan A. Strauss S. Tofanelli R. Mody T. A. Lee K. Tsiantis M.. (2022). The annotation and analysis of complex 3D plant organs using 3DCoordX. Plant Physiol. 189, 1278–1295. doi: 10.1093/plphys/kiac145 35348744 PMC9237718

[B102] Vijayan A. Tofanelli R. Strauss S. Cerrone L. Wolny A. Strohmeier J.. (2021). A digital 3D reference atlas reveals cellular growth patterns shaping the Arabidopsis ovule. eLife 10, e63262. doi: 10.7554/eLife.63262 33404501 PMC7787667

[B103] Wagner D. Sablowski R. W. M. Meyerowitz E. M. (1999). Transcriptional activation of APETALA1 by LEAFY. Science 285, 582–584. doi: 10.1126/science.285.5427.582 10417387

[B104] Wagner D. Wellmer F. Dilks K. William D. Smith M. R. Kumar P. P.. (2004). Floral induction in tissue culture: a system for the analysis of LEAFY-dependent gene regulation. Plant J. 39, 273–282. doi: 10.1111/j.1365-313X.2004.02127.x 15225291

[B105] Waylen L. N. Nim H. T. Martelotto L. G. Ramialison M. (2020). From whole-mount to single-cell spatial assessment of gene expression in 3D. Commun. Biol. 3, 1–11. doi: 10.1038/s42003-020-01341-1 33097816 PMC7584572

[B106] Weigel D. Alvarez J. Smyth D. R. Yanofsky M. F. Meyerowitz E. M. (1992). LEAFY controls floral meristem identity in Arabidopsis. Cell 69, 843–859. doi: 10.1016/0092-8674(92)90295-N 1350515

[B107] Wellmer F. Alves-Ferreira M. Dubois A. Riechmann J. L. Meyerowitz E. M. (2006). Genome-wide analysis of gene expression during early arabidopsis flower development. PloS Genet. 2, e117. doi: 10.1371/journal.pgen.0020117 16789830 PMC1523247

[B108] Whittaker C. Dean C. (2017). The FLC locus: A platform for discoveries in epigenetics and adaptation. Annu. Rev. Cell Dev. Biol. 33, 555–575. doi: 10.1146/annurev-cellbio-100616-060546 28693387

[B109] Wu H.-W. Deng S. Xu H. Mao H.-Z. Liu J. Niu Q.-W.. (2018). A noncoding RNA transcribed from the AGAMOUS (AG) second intron binds to CURLY LEAF and represses AG expression in leaves. New Phytol. 219, 1480–1491. doi: 10.1111/nph.15231 29862530

[B110] Wu M.-F. Sang Y. Bezhani S. Yamaguchi N. Han S.-K. Li Z.. (2012). SWI2/SNF2 chromatin remodeling ATPases overcome polycomb repression and control floral organ identity with the LEAFY and SEPALLATA3 transcription factors. Proc. Natl. Acad. Sci. 109, 3576–3581. doi: 10.1073/pnas.1113409109 22323601 PMC3295252

[B111] Wu M.-F. Wagner D. (2012). “RNA *in situ* hybridization in arabidopsis,” in RNA abundance analysis: methods and protocols methods in molecular biology. Eds. Jin H. Gassmann W. (Totowa, NJ: Humana Press), 75–86. doi: 10.1007/978-1-61779-839-9_5 22589125

[B112] Xu X. Smaczniak C. Muino J. M. Kaufmann K. (2021). Cell identity specification in plants: lessons from flower development. J. Exp. Bot. 72, 4202–4217. doi: 10.1093/jxb/erab110 33865238 PMC8163053

[B113] Xu Y. Yamaguchi N. Gan E.-S. Ito T. (2019). When to stop: an update on molecular mechanisms of floral meristem termination. J. Exp. Bot. 70, 1711–1718. doi: 10.1093/jxb/erz048 30916342

[B114] Yadav R. K. Girke T. Pasala S. Xie M. Reddy G. V. (2009). Gene expression map of the Arabidopsis shoot apical meristem stem cell niche. Proc. Natl. Acad. Sci. 106, 4941–4946. doi: 10.1073/pnas.0900843106 19258454 PMC2660727

[B115] Yadav R. K. Tavakkoli M. Xie M. Girke T. Reddy G. V. (2014). A high-resolution gene expression map of the Arabidopsis shoot meristem stem cell niche. Development 141, 2735–2744. doi: 10.1242/dev.106104 24961803

[B116] Yamaguchi N. (2021). LEAFY, a pioneer transcription factor in plants: A mini-review. Front. Plant Sci. 12. doi: 10.3389/fpls.2021.701406 PMC8287900 34290727

[B117] Yamaguchi N. Huang J. Tatsumi Y. Abe M. Sugano S. S. Kojima M.. (2018). Chromatin-mediated feed-forward auxin biosynthesis in floral meristem determinacy. Nat. Commun. 9, 5290. doi: 10.1038/s41467-018-07763-0 30538233 PMC6289996

[B118] Yamaguchi N. Huang J. Xu Y. Tanoi K. Ito T. (2017). Fine-tuning of auxin homeostasis governs the transition from floral stem cell maintenance to gynoecium formation. Nat. Commun. 8, 1125. doi: 10.1038/s41467-017-01252-6 29066759 PMC5654772

[B119] Yamaguchi N. Wu M.-F. Winter C. M. Berns M. C. Nole-Wilson S. Yamaguchi A.. (2013). A molecular framework for auxin-mediated initiation of flower primordia. Dev. Cell 24, 271–282. doi: 10.1016/j.devcel.2012.12.017 23375585

[B120] Yanofsky M. F. Ma H. Bowman J. L. Drews G. N. Feldmann K. A. Meyerowitz E. M. (1990). The protein encoded by the Arabidopsis homeotic gene agamous resembles transcription factors. Nature 346, 35–39. doi: 10.1038/346035a0 1973265

[B121] Yin R. Xia K. Xu X. (2023). Spatial transcriptomics drives a new era in plant research. Plant J., n/a. doi: 10.1111/tpj.16437 37651723

[B122] Yosef N. Regev A. (2011). Impulse control: temporal dynamics in gene transcription. Cell 144, 886–896. doi: 10.1016/j.cell.2011.02.015 21414481 PMC3148525

[B123] Yu X. Liu Z. Sun X. (2022). Single-cell and spatial multi-omics in the plant sciences: Technical advances, applications, and perspectives. Plant Commun. 4, 100508. doi: 10.1016/j.xplc.2022.100508 36540021 PMC10203395

[B124] Zaret K. S. Carroll J. S. (2011). Pioneer transcription factors: establishing competence for gene expression. Genes Dev. 25, 2227–2241. doi: 10.1101/gad.176826.111 22056668 PMC3219227

[B125] Zhang D. Deng Y. Kukanja P. Agirre E. Bartosovic M. Dong M.. (2023). Spatial epigenome–transcriptome co-profiling of mammalian tissues. Nature 616, 113–122. doi: 10.1038/s41586-023-05795-1 36922587 PMC10076218

