# Supplementary material for: Old school, new rules: floral meristem development revealed by 3D gene expression atlases and high-resolution transcription factor–chromatin dynamics
Source: Front Plant Sci. 2023 Dec 13;14:1323507. doi: 10.3389/fpls.2023.1323507 (PMC10753784; doi:10.3389/fpls.2023.1323507)
Supplement: Supplementary file 1 [file Table_1.docx]

Table 1. List of 28 genes curated in the 4D multiscale FM atlas.

| Gene name | AGI code |
| --- | --- |
| *AGAMOUS* (*AG*) | AT4G18960 |
| *AHP6/HISTIDINE PHOSPHOTRANSFER PROTEIN 6* | AT1G80100 |
| *AINTEGUMENTA* (*ANT*) | AT4G37750 |
| *APETALA1* (*AP1*) | AT1G69120 |
| *APETALA2* (*AP2*) | AT4G36920 |
| *APETALA3* (*AP3*) | AT3G54340 |
| *ASYMMETRIC LEAVES 1* (*AS1*) | AT2G37630 |
| *MERISTEM LAYER 1* (*ATML1*) | AT4G21750 |
| *CLAVATA3* (*CLV3*) | AT2G27250 |
| *CUP-SHAPED COTYLEDON1* (*CUC1*) | AT3G15170 |
| *CUP-SHAPED COTYLEDON2* (*CUC2*) | AT5G53950 |
| *CUP-SHAPED COTYLEDON3* (*CUC3*) | AT1G76420 |
| *AUXIN RESPONSE TRANSCRIPTION FACTOR 3* (*ETTIN/ARF3*) | AT2G33860 |
| *FILAMENTOUS FLOWER* (*YABBY1*) | AT2G45190 |
| *LEAFY* (*LFY*) | AT5G61850 |
| *MONOPTEROS* (*MP*) | AT1G19850 |
| *PHABULOSA* (*PHB*) | AT2G34710 |
| *PHAVOLUTA* (*PHV*) | AT1G30490 |
| *PISTILLATA* (*PI*) | AT5G20240 |
| *PUCHI* | AT5G18560 |
| *REVOLUTA* (*REV*) | AT5G60690 |
| *SEPALLATA1/AGL2* (*SEP1*) | AT5G15800 |
| *SEPALLATA2* (*SEP2*) | AT3G02310 |
| *SEPALLATA3/AGL9* (*SEP3*) | AT1G24260 |
| *SHOOTMERISTEMLESS* (*STM*) | AT1G62360 |
| *SUPERMAN* (*SUP*) | AT3G23130 |
| *SHORT VEGETATIVE PHASE* (*SVP*) | AT2G22540 |
| *WUSCHEL* (*WUS*) | AT2G17950 |

Table 2. Gene combinations expressed in ‘cell states’ from floral stages 0 to 4 identified in the 4D multiscale FM atlas (Refahi et al., 2021).

| Cell state | Expressed genes |
| --- | --- |
| 1 | *ANT, AS1, ATML1, ETTIN, FIL, LFY, MP* |
| 2 | *ANT, ATML1, ETTIN, LFY, MP, PHB, PHV, PUCHI, REV* |
| 3 | *ANT, ATML1, LFY, MP, PHB, PHV, PUCHI, REV* |
| 4 | *ATML1, CUC1, CUC2, CUC3, MP, STM* |
| 5 | *ANT, AP1, AP2, AS1, ATML1, ETTIN, FIL, LFY, MP, SVP* |
| 6 | Not specified |
| 7 | *ANT, AP1, AP2, ATML1, LFY, MP, PHB, PHV, REV, STM, SVP* |
| 8 | *AHP6, ANT, AP1, AP2, ATML1, ETTIN, LFY, MP, SEP1, SEP2* |
| 9 | *ANT, AP1, AP2, AS1, ATML1, ETTIN, FIL, LFY, SEP1, SEP2, STM, SVP* |
| 10 | *ANT, AP1, AP2, ATML1, ETTIN, LFY, MP, PHB, PHV, REV, SEP1, SEP2, STM* |
| 11 | *ANT, AP1, AP2, ATML1, ETTIN, LFY, MP, SEP1, SEP2,* |
| 12 | *ANT, AP1, AP2, ATML1, ETTIN, LFY, MP, SEP1, SEP2, STM* |
| 13 | *AG, AP3, ATML1, ETTIN, MP, PHB, PHV, PI, REV, SEP1, SEP2, SEP3, STM* |
| 14 | *AG, ATML1, CLV3, ETTIN, MP, PHB, PHV, REV, SEP1, SEP2, SEP3, STM* |
| 15 | *AG, ATML1, ETTIN, MP, PHB, PHV, REV, SEP1, SEP2, SEP3, STM* |
| 16 | *AHP6, ANT, AP1, AP2, AS1, ATML1, ETTIN, FIL, LFY, MP, SEP1, SEP2* |
| 17 | *ANT, AP1, AP2, AS1, ATML1, ETTIN, FIL, LFY, MP, SEP1, SEP2* |
| 18 | *ANT, AP1, AP2, AS1, ATML1, LFY, MP, PHB, PHV, REV, SEP1, SEP2* |
| 19 | *AP1, AP2, AP3, ATML1, CUC1, CUC2, CUC3, MP, PHB, PHV, PI, REV, SEP1, SEP2, STM* |
| 20 | *AP1, AP2, ATML1, CUC1, CUC2, CUC3, MP, SEP1, SEP2, STM* |
| 21 | Not specified |
| 22 | *AG, ANT, AP3, ATML1, ETTIN, MP, PHB, PHV, PI, SEP1, SEP2, SEP3, STM* |
| 23 | *AG, ANT, ATML1, ETTIN, MP, PHB, PHV, REV, SEP1, SEP2, SEP3, STM* |
| 24 | *AG, ANT, ATM1, ETTIN, MP, PHB, PHV, REV, SEP1, SEP2, SEP3, STM, SUP* |
| 25 | *AG, AP3, ATM1, CUC1, CUC2, CUC3, MP, PI, SEP1, SEP2, SEP3, STM* |
| 26 | *AHP6, ANT, AP1, AP2, AS1, ATML1, LFY, MP, PHB, PHV, REV, SEP1, SEP2* |
| 27 | *ANT, AP1, AP2, AS1, ATML1, ETTIN, FIL, LFY, SEP1, SEP2* |
| 28 | Not specified |
| 29 | *AP1, AP2, AP3, ATML1, CUC1, CUC2, CUC3, MP, PI, SEP1, SEP2, STM* |
| 30 | *AP1, AP2, AP3, ATML1, CUC1, CUC2, CUC3, MP, SEP1, SEP2, STM* |
| 31 | *ANT, AS1, ATML1, ETTIN, FIL, LFY, MP, PUCHI* |
